# West Nile virus triggers intestinal dysmotility via T cell–mediated enteric nervous system injury

**DOI:** 10.1172/JCI181421

**Published:** 2024-08-29

**Authors:** Hana Janova, Fang R. Zhao, Pritesh Desai, Matthias Mack, Larissa B. Thackray, Thaddeus S. Stappenbeck, Michael S. Diamond

**Affiliations:** 1 Department of Medicine, Washington University School of Medicine, Saint Louis, Missouri, USA.; 2 Department of Nephrology, University Hospital Regensburg, Regensburg, Germany.; 3 Department of Inflammation and Immunity, Cleveland Clinic, Cleveland, Ohio, USA.; 4 Department of Pathology and Immunology,; 5 Department of Molecular Microbiology, and; 6 The Andrew M. and Jane M. Bursky Center for Human Immunology and Immunotherapy Programs, Washington University School of Medicine, St. Louis, Missouri, USA.

**Keywords:** Gastroenterology, Infectious disease, Fas signaling, Neurological disorders, T cells

## Abstract

Intestinal dysmotility syndromes have been epidemiologically associated with several antecedent bacterial and viral infections. To model this phenotype, we previously infected mice with the neurotropic flavivirus West Nile virus (WNV) and demonstrated intestinal transit defects. Here, we found that within 1 week of WNV infection, enteric neurons and glia became damaged, resulting in sustained reductions of neuronal cells and their networks of connecting fibers. Using cell-depleting antibodies, adoptive transfer experiments, and mice lacking specific immune cells or immune functions, we show that infiltrating WNV-specific CD4^+^ and CD8^+^ T cells damaged the enteric nervous system (ENS) and glia, which led to intestinal dysmotility; these T cells used multiple and redundant effector molecules including perforin and Fas ligand. In comparison, WNV-triggered ENS injury and intestinal dysmotility appeared to not require infiltrating monocytes, and damage may have been limited by resident muscularis macrophages. Overall, our experiments support a model in which antigen-specific T cell subsets and their effector molecules responding to WNV infection direct immune pathology against enteric neurons and supporting glia that results in intestinal dysmotility.

## Abstract 2

Antecedent gastrointestinal infections are thought to trigger intestinal dysmotility. We show how responding T cells to a viral infection have adverse consequences for intestinal motility.

## Graphical abstract

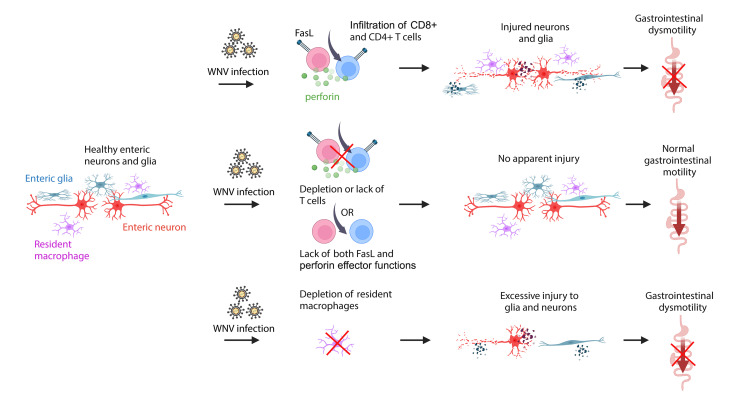

## Introduction

Properly regulated intestinal motility allows for efficient timing of nutrient uptake and elimination of waste. Peristalsis of the intestines is regulated primarily by the peripheral enteric nervous system (ENS) with neural inputs from the central nervous system (CNS) (1). The neuronal bodies of the ENS are concentrated in ganglia and embedded in 2 interconnected plexi: the submucosal plexus in the submucosa and the myenteric plexus, positioned between the inner circular and outer longitudinal smooth muscle layers of the intestine. Enteric neurons function with other cells including glia, interstitial cells of Cajal (ICCs), and muscularis macrophages to regulate intestinal motility. Injury, dysfunction, or depletion of any of these cells can result in intestinal dysmotility (2–8).

It is estimated that more than 40% of adults suffer at some point during their lifetime from some form of chronic gastrointestinal (GI) motility disorder that negatively affects quality of life (9, 10). Although age, sex, genetics, diet, and socioeconomic factors are associated with the development of these disorders, antecedent GI tract infections with bacteria or viruses are thought to have roles in triggering intestinal dysmotility (11). Despite the prevalence of GI tract motility disorders, components of the underlying mechanisms of injury have been established experimentally in only a few instances. In mice, enteric infection with *Salmonella typhimurium* bacteria triggers long-term GI tract dysmotility due to loss of neurons via caspase-1– and caspase-11–dependent apoptosis (12). Systemic infection of mice with herpes simplex virus 1 (HSV-1) causes acute injury to enteric neurons and glia through a mechanism requiring neutrophils or macrophages that produce reactive oxygen and nitrogen species (13, 14). West Nile virus (WNV), a mosquito-transmitted flavivirus, can infect neurons in the small intestine after subcutaneous inoculation and viremic spread to cause acute and relapsing GI tract dysmotility (2).

Although WNV infection damages the ENS (2), the mechanisms and targets of injury remain poorly understood. Here, we show that WNV infection caused injury to the ENS and glial networks. Although infiltrating monocytes and monocyte-derived macrophages in the myenteric plexus and the muscular layers of the small intestine were present at multiple time points after WNV infection, genetic depletion or inhibition of their recruitment did not prevent damage to the neuronal network or affect WNV-triggered GI dysmotility. Rather, using multiple transgenic mouse strains and by performing immune cell depletions and adoptive transfer studies, we showed that WNV-specific CD4^+^ and CD8^+^ T cells used several different effector mechanisms to damage enteric neurons and neighboring glial cells, leading to GI transit dysfunction. Moreover, this T cell–mediated injury was worsened by the depletion of resident muscularis macrophages. Overall, our study defines how both resident and infiltrating innate and adaptive immune cells in the GI tract respond to viral infection to reshape the ENS architecture with consequences for intestinal motility.

## Results

### WNV infection triggers persistent changes in the enteric neuronal network.

**Figure 1:**
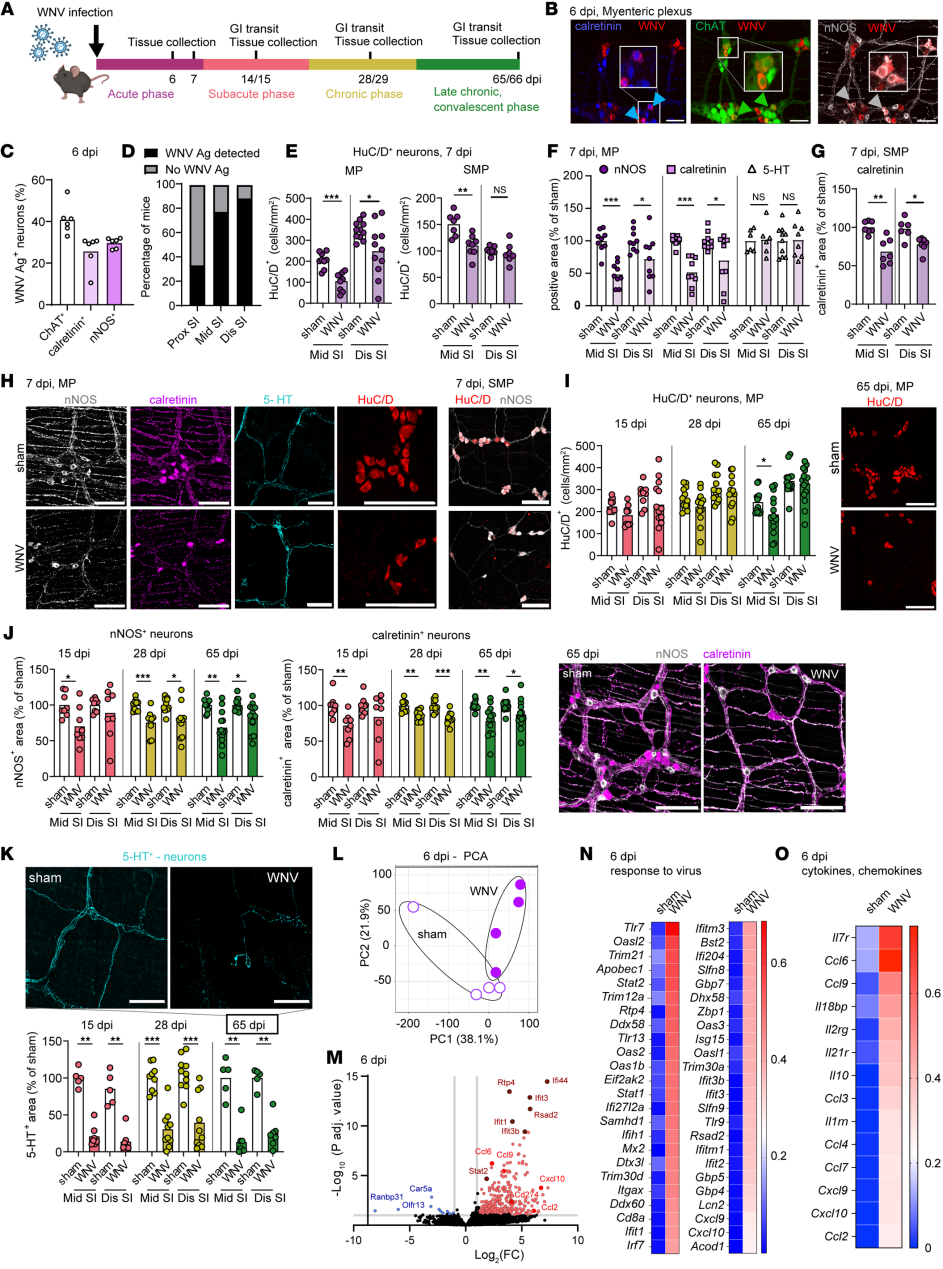
WNV infection induces changes in ENS neuronal networks. (**A**) Nine- to 10-week-old C57BL6/J male mice were inoculated in the footpad with 10^2^ FFU WNV (New York 1999 strain), and a carmine dye transit assay and tissue collections were done at the indicated time points. The figure in **A** was created using BioRender software. (**B** and **C**) Whole-mount preparations of the muscularis externa from *ChAT-*eGFP reporter mice were isolated at 5 or 6 dpi and costained for WNV antigen, calretinin^+^, and nNOS^+^ neurons. (**B**) Blue, green, and white arrowheads indicate WNV antigen^+^ calretinin^+^ neurons, ChAT^+^ neurons, and nNOS^+^ neurons, respectively. Images are representative of 2 experiments. Scale bars: 50 μm. (**C**) Proportion of neuronal subgroups infected with WNV. (**D**) Percentage of mice that had WNV antigen (Ag) in the proximal (Prox), middle (Mid), and distal (Dis) regions of the small intestine (SI) at 6 dpi. (**E**–**K**) The muscularis externa with the attached layer containing the submucosal plexus (SMP) (**G**), the myenteric plexus (MP) (**F** and **I**–**K**), or both the myenteric plexus and submucosal plexus, as indicated (**E** and **H**), was isolated from the middle and distal small intestine of sham- and WNV-infected mice at 7 dpi (**E**–**H**) or at 15, 28, and 65 dpi (**I**–**K**) and stained for neuronal markers. (**E** and **I**) The total number of HuC/D^+^ neurons in (**E**) the submucosal plexus and myenteric plexus and (**I**) the myenteric plexus only was counted and is shown as the number of neurons per mm^2^. (**F** and **G**) The fraction of area that stained positively for nNOS, calretinin, and 5-HT in the myenteric plexus (**F**) or for calretinin in the submucosal plexus (**G**); values were normalized to those for sham-infected mice. Circles, squares, and triangles indicate nNOS^+^, calretinin^+^, and 5-HT^+^ neurons, respectively. (**H**) Images show staining for the indicated markers in the middle small intestine from sham- and WNV-infected mice at 7 dpi in either the myenteric plexus or the submucosal plexus. Scale bars: 100 μm. (**J**) nNOS^+^ and calretinin^+^ and (**K**) 5-HT^+^ cell areas; values were normalized to those for sham-infected mice. (**I**–**K**) Images show staining in the middle small intestine from sham- and WNV-infected mice at 65 dpi. Scale bars: 100 μm. (**L**–**O**) Analysis of neuron-specific RNA-Seq using TRAP in WNV- or mock-infected *Snap25l10a* mice at 6 dpi. (**L**) Principal component analysis (PCA). (**M**) Volcano plot of differential expression analysis (DEseq2) of translating ribosome affinity purification sequencing (TRAP-Seq) comparing WNV- and mock-infected samples. Red dots indicate a log_2_ fold change of greater than 1 and a FDR (*P*-adjusted) of less than 0.05, and blue dots indicate a log_2_ fold change of 1 or less and a *P*-adjusted FDR of less than 0.05. (**N** and **O**) Heatmap of differentially expressed genes in sham- and WNV-infected mice showing genes related to (**N**) the response to virus and (**O**) cytokines and chemokines. Expression levels were normalized across each gene and represent the average of 4 mice per condition. Data were pooled from the following number of experiments: (**C** and **D**) 2; (**E**–**K**) 3 (myenteric plexus) and 2 (submucosal plexus); (**F**) 3; (**G**) 2, (**I**) 3; (**J**) 2 (15 dpi), 3 (28 dpi), and 4 (65 dpi); and (**K**) 2 (15 dpi), 3 (28 dpi), and 2 (65 dpi). The numbers of mice per group were as follows: (**C**) *n* = 6; (**D**) *n* = 9; (**E**) *n* = 7–11; (**F**) *n* = 6–10; (**G**) *n* = 6–7; (**I**) *n* = 9–16; (**J**) *n* = 8–13; (**K**) *n* = 5–10. Column heights in **C**, **E**–**G**, and **I**–**K** indicate mean values. *P < 0.05, **P < 0.01, and ***P < 0.001, by 2-tailed Mann-Whitney *U* test.

Our previous work described a dysmotility syndrome after WNV infection with preferential effects in the small intestine (2). WNV antigen localizes to enteric neurons of the small intestine during the acute phase of infection (at 6 days post infection [dpi]) after subcutaneous inoculation (2); however, the viral tropism for specific neuronal cell populations is unclear. To address this question, we inoculated *ChAT-eGFP* reporter mice, which identify cholinergic neurons, with WNV (Figure 1A) and costained whole-mount tissue preparations of small intestines with antisera against WNV (15), as well as for other neuronal cell subsets. WNV antigen was detected at similar percentages in calretinin^+^, ChAT^+^, and nNOS^+^ neurons (Figure 1, B and C, and Supplemental Figure 1, A–C). WNV antigen in the myenteric and submucosal plexuses varied along the length of the small intestine, with the middle (jejunal) and distal (ileal) segments showing the highest penetrance (~70%–90% of mice) and the proximal (duodenal) region having less penetrance (20%–33% of mice) (Figure 1D and Supplemental Figure 1D). Thus, we focused subsequent analyses on the middle and distal regions of the small intestine.

To assess the effect of WNV infection on the small intestine, we first quantified the number of neuronal bodies in whole-mount tissue preparations by staining for HuC/D, a pan-neuronal marker. In the myenteric plexus, we found that neuronal cell body numbers were decreased in the middle and distal regions of the small intestine at 7 dpi compared with mock-infected controls (Figure 1, E and H). Similarly, we observed lower numbers of HuC/D^+^ neurons in the submucosal plexus of the middle region of the small intestine (Figure 1E). The loss of neuronal bodies within intestinal ganglia after WNV infection was associated with a decreased density of their axonal fiber networks (referred to herein as neuronal network density) in the myenteric plexus and the inner circular muscle layer of the muscularis propria in the middle and distal regions of the small intestine at 7 dpi; this included a reduction of the pan-neuronal peripherin^+^ network and of calretinin^+^ and nNOS^+^ networks that are major neuronal cell subsets in the myenteric plexus and inner circular muscle layer (Figure 1, F and H, and Supplemental Figure 1, E–H). In contrast, the density of serotonergic myenteric neuronal networks (i.e., those secreting 5-hydroxytryptamine [5-HT]) in the myenteric plexus at 7 dpi was not different from that of sham-infected controls (Figure 1, F and H). In the submucosal plexus, the calretinin^+^ neuronal network density was also diminished with WNV infection, which corresponded to the decreased numbers of neurons (Figure 1, G and H). However, as submucosal plexus neurons do not contribute substantially to GI motility (16), we focused on the neuronal cells and networks in the myenteric plexus.

As dysmotility after WNV infection can persist through 65 days (Supplemental Figure 1I and (2)), we quantified the number of HuC/D^+^ neurons and assessed the neuronal network density in the myenteric plexus at later post-infection time points: 15 dpi (subacute phase), 28 dpi (chronic phase), and 65 dpi (late chronic, convalescent phase) (Figure 1A). By 65 dpi, the numbers of HuC/D^+^ neurons in the distal small intestine showed near-complete recovery, a process that began as early as 15 dpi (Figure 1I). However, this recovery did not occur to the same extent in the middle region of the small intestine, as fewer neurons were detected at 65 dpi than in sham-infected controls. Our analysis of neuronal networks showed a durable loss of the density of nNOS^+^ and calretinin^+^ neuronal networks (Figure 1J) at 15, 28, and 65 dpi in both the middle and distal regions of the small intestine. In contrast, we detected no differences in neuronal network density in the proximal region of the small intestine at 28 dpi, a region that had less viral antigen detected at 6 dpi (Supplemental Figure 1, C and L). Although skewing of neuronal cell subgroup proportions can affect GI motility (7, 17), we did not detect differences in the proportions of nNOS^+^ or calretinin^+^ neurons at any point after WNV infection (Supplemental Figure 1, J and K). However, WNV infection caused a marked loss in the density of 5-HT^+^ neuronal processes at 15, 28, and 65 dpi in the middle and distal small intestine (Figure 1K). This neuronal cell subset is important for the formation of new neurons after intestinal injury (18, 19).

To identify factors that might regulate the ENS response to WNV infection in the acute phase (6 dpi), we performed a translating ribosomal affinity purification (TRAP) of the muscularis externa of WNV- and mock-infected *Snap25l10a* GFP mice. *Snap25l10a* GFP mice express a GFP-tagged ribosomal subunit 10la in all neurons, which enables isolation of RNA predominantly from neurons (Supplemental Figure 1M). RNA-Seq showed increased expression of antiviral genes and pathways (e.g., IFN-stimulated genes [ISGs], *Ifit* family members, *Stat1/2* pathways, and pattern recognition receptor signaling pathways) (Figure 1, L–N, and Supplemental Figure 1N). We also observed higher levels of mRNAs encoding *Ccl6*, *Ccl3*, *Cxcl10*, and *Ccl2* cytokines, which stimulate chemotaxis of T cells, monocytes, and macrophages (Figure 1O). Furthermore, WNV infection led to an increase in transcripts associated with antigen presentation, including components of MHC class I (*Tap1*, *B2m*) and MHC class II (*H2-DMb1*) antigen processing (Supplemental Figure 1, O and P). These RNA-Seq data identified gene signatures in a neuron-enriched population from the muscularis externa with possible antiviral, immune cell trafficking, and immunomodulatory effects in response to viral infection.

### WNV infection triggers persistent changes in intestinal glial cell networks.

**Figure 2:**
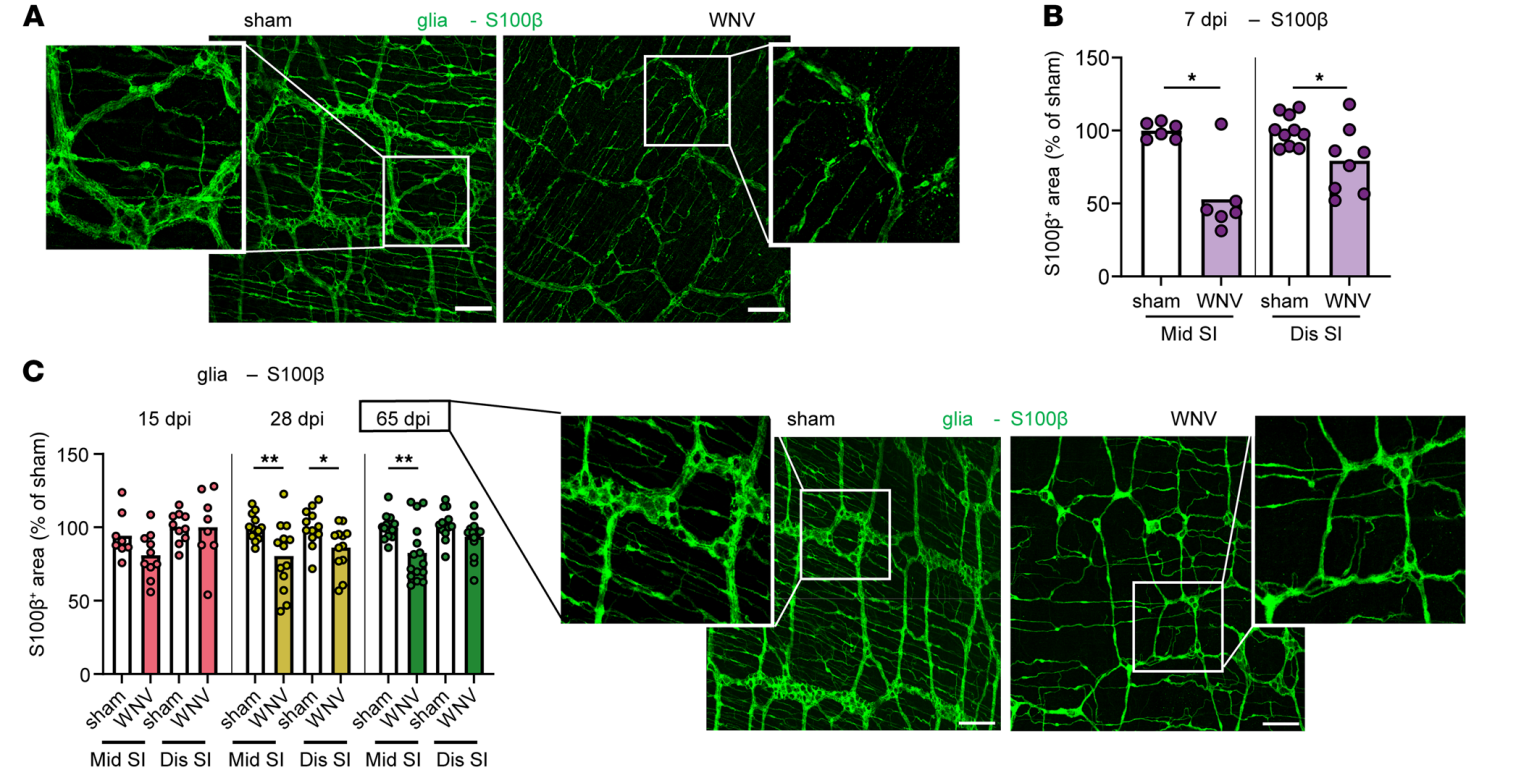
WNV infection affects enteric glial networks. (**A**–**C**) The muscularis externa was isolated from middle and distal regions of the small intestine of sham- or WNV-infected C57BL/6J mice at (**A** and **B**) 7 dpi or (**C**) 15, 28, and 65 dpi and stained for glia (S100β). The fraction of area that stained positive for S100β was determined, and the values were normalized to sham-infected mice. Representative images show S100β staining in the middle region of the small intestine in sham- and WNV-infected mice at (**A**) 7 dpi or (**C**) 65 dpi. (**A** and **C**) Scale bars: 100 μm. Original magnification, ×2.5 (enlarged insets). Data were pooled from (**A** and **B**) 2 experiments (*n =* 5–10) and (**C**) (left to right) 2, 3, and 4 experiments (*n =* 6–10, 12–13, and 13–16). Column heights indicate the mean values. *P < 0.05 and **P < 0.01, by 2-tailed Mann-Whitney *U* test.

Enteric glia provide structural and metabolic support for enteric neurons and contribute to neurogenesis (20–25). To determine whether the glial network is affected by WNV infection, we evaluated intestinal whole mounts from WNV-infected mice at 7 dpi by coimmunostaining with antisera against WNV and antibodies against the pan-glial marker S100β (20). Despite limited detection of WNV-antigen^+^ glial cells (Supplemental Figure 2A), the S100β^+^ glial network density in the myenteric plexus was markedly diminished in WNV-infected mice at 7 dpi (Figure 2, A and B), with sustained reductions also observed at 28 and 65 dpi (Figure 2C). However, not all neuronal cell–associated networks in the ENS showed diminished density following WNV infection. ICCs located in the smooth muscle layer act as transducers of signals from enteric neurons to smooth muscle cells (8). Although WNV antigen localized sporadically to ICCs in the circular muscle layer of the small intestine at 6 dpi (Supplemental Figure 2B), their density, as judged by cKit staining (26), was like that in sham-treated mice at 15, 28, or 65 dpi (Supplemental Figure 2C).

### Monocyte and macrophage infiltration is not required for ENS damage or intestinal dysmotility following WNV infection.

**Figure 3:**
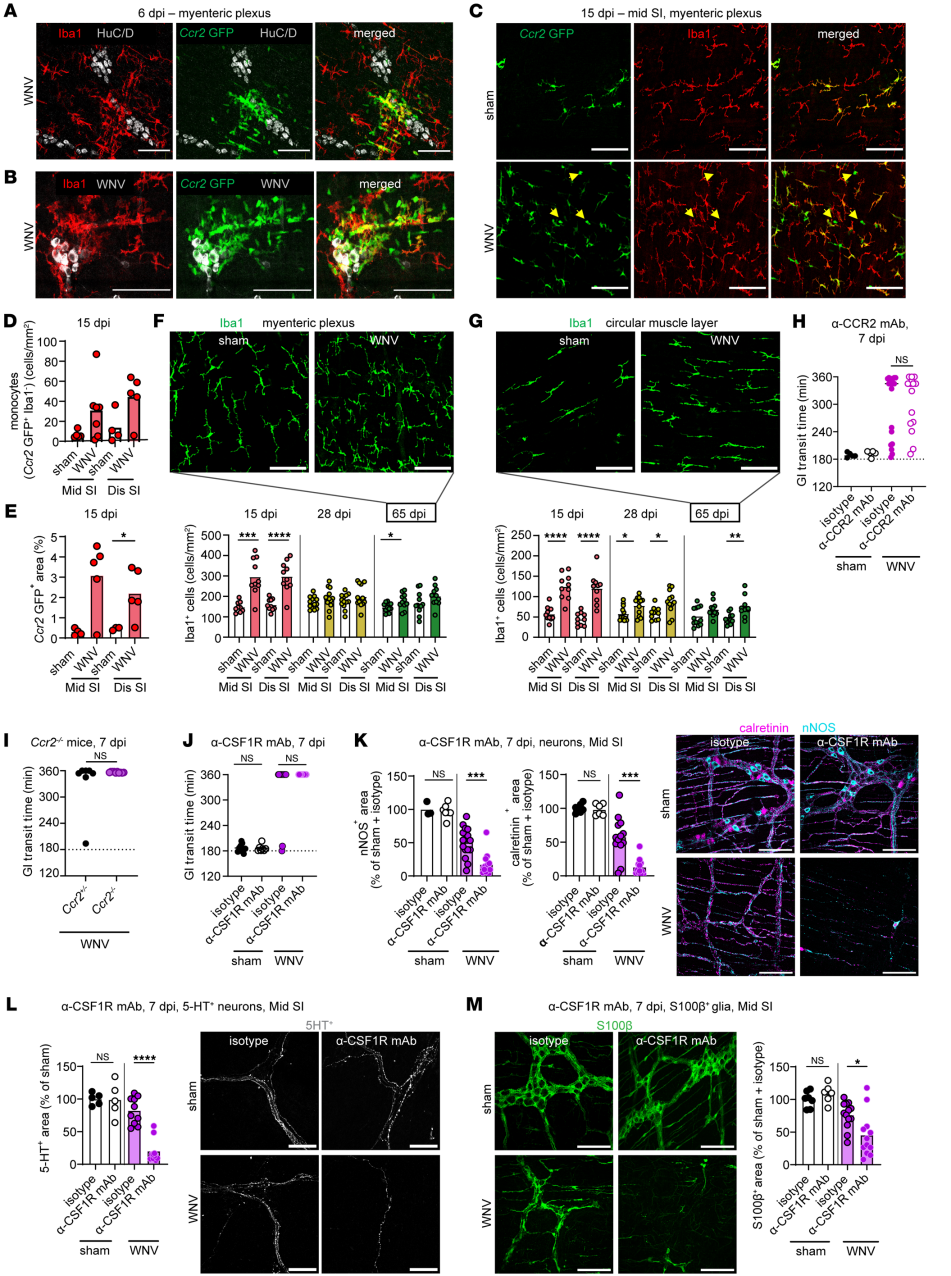
WNV infection promotes infiltration of monocytes into the intestine. (**A**–**E**) Whole-mount preparations of the muscularis externa were isolated from the middle and distal regions of the small intestine from WNV-infected heterozygous *Ccr2-*GFP mice at (**A** and **B**) 6 or (**C**) 15 dpi and stained for (**A**) neuron (HuC/D) and macrophage (Iba1) markers, (**B**) WNV antigen and macrophage markers, or (**C**–**E**) macrophage markers. Yellow arrowheads indicate monocytes (CCR2 GFP^+^Iba1^–^ cells). Scale bars: 100 μm. (**A**–**C**) Images were obtained from the myenteric plexus of the middle region of the small intestine from at least 2 experiments. (**D**) Monocytes (*Ccr2* GFP^+^Iba1^–^) in the myenteric plexus are shown as the numbers of cells per mm^2^. (**E**) The fraction of *Ccr2* GFP^+^ area (representing monocytes and/or monocyte-derived macrophages) in the myenteric plexus of WNV- or sham-infected mice. (**F** and **G**) Muscularis externa of the middle and distal small intestines from sham- or WNV-infected mice harvested at 15, 28, or 65 dpi were stained for Iba1^+^ macrophages. Macrophages in (**F**) the myenteric plexus and (**G**) the circular muscle layer are shown as the number of Iba1^+^ cells per mm^2^. Images of Iba1 staining in sham- or WNV-infected mice at 65 dpi. Scale bars: 100 μm. (**H**–**J**) GI transit was measured after oral gavage of carmine red dye (**H**) in sham- or WNV-infected mice (at 7 dpi) after treatment with anti-CCR2 or isotype mAbs (**I**) in WNV-infected *Ccr2^+/–^* and *Ccr2^–/–^* mice, and (**J**) in sham- or WNV-infected mice after treatment with anti-CSF1R or an isotype control mAb. (**K**–**M**). Whole-mount preparations of the muscularis externa were isolated from the middle region of small intestine of WNV-infected mice treated with anti-CSF1R or isotype mAbs and stained for (**K**) nNOS^+^ and calretinin^+^ neurons, (**L**) 5-HT^+^ neurons, or (**M**) S100β^+^ glia. Scale bars: 100 μm. The fraction of the area that stained positive for calretinin, nNOS, 5-HT, or S100β; values were normalized to those for sham-infected mice treated with an isotype control mAb. Data were pooled from (**D** and **E**) 2; (**F** and **G**) 2 (15 dpi); 3 (28 dpi) and 4 (65 dpi); (**H**) 3; (**I**) 1; (**J**) 3; and (**K**–**M**) 3 experiments. The numbers of mice per group were as follows: (**D** and **E**) *n* = 4–7; (**F**) *n* = 9–13; (**G**) *n* = 10–12; (**H**) *n* = 5–20; (**I**) *n* = 7–10; (**J**) *n* = 7–16; (**K**) *n* = 6–13; (**L**) *n* = 5–10; and (**M**) *n* = 7–13. Column heights in **D**–**G** and **J**–**L** indicate mean values, and lines in **H**–**J** indicate median values. *P < 0.05, **P < 0.01, ***P < 0.001, and ****P < 0.0001, by (**D**, **F** and **G**) 2-tailed Mann-Whitney *U* test and (**L**) Kruskal-Wallis ANOVA with Dunn’s post test.

Monocytes and monocyte-derived macrophages have been shown to injure the intestine in the context of herpesvirus infection (14, 27). Given our data showing higher levels of myeloid cell chemoattractant mRNAs (e.g., *Ccl2*, *Ccl3*, *Ccl6*, and *Ccl9*) in the muscularis externa of WNV-infected mice (Figure 1O), we quantified monocyte accumulation at 6 dpi in the small intestine of WNV-infected mice using heterozygous *Ccr2*-GFP reporter mice after staining whole-mount preparations for Iba1, a marker of monocyte-derived macrophages and endogenous muscularis macrophages, but not recent monocyte immigrants (28, 29). The number of both monocytes (*Ccr2*-GFP^+^Iba1^–^ cells) and macrophages (*Ccr2*-GFP^+^Iba1^+^ cells) was increased in the proximity of WNV-infected neurons at 6 dpi; the elevation of monocytes persisted up to 15 dpi in the myenteric plexus and the circular inner smooth muscle layer of the muscularis externa (Figure 3, A–E). Elevated numbers of macrophages (Iba1^+^ cells) persisted through 65 dpi, with a peak at 15 dpi (Figure 3, C, F, and G). Fate-mapping studies using *Ccr2* CreER YFP reporter mice demonstrated that the increased number of macrophages was due to infiltrating monocytes, since most of the Iba1^+^ cells also expressed YFP (Supplemental Figure 3A).

To assess the role of the infiltrating monocytes in delaying GI transit in WNV-infected mice, we inhibited their migration into the intestines using an anti-CCR2–blocking mAb (30) or *Ccr2^–/–^* mice (Supplemental Figure 3, B and C). We found that reduced accumulation of monocytes during acute WNV infection did not prevent damage to neuronal or glial networks at 7 dpi and did not affect the delayed GI transit time phenotype at 7 or 15 dpi (Figure 3, H and I, and Supplemental Figure 3, D–G). Neutrophil infiltration causes injury to enteric neurons and GI dysmotility after herpesvirus infection in mice (13) and can contribute to WNV-induced pathogenesis in the brain (31–33). However, complete and partial depletion of neutrophils and monocytes, respectively, with an anti-Ly6G/anti-Ly6C mAb (Gr-1) did not improve WNV-induced GI tract dysmotility at 7 dpi (Supplemental Figure 3, B and H–J). We confirmed this result by injecting *Ccr2^–/–^* mice with anti-Ly6G/anti-Ly6C mAb to ensure that a lack of monocyte infiltration during WNV infection was not compensated by an increased influx of neutrophils (34) (Supplemental Figure 3, B, K, and L). Thus, WNV-induced intestinal dysmotility appeared to be independent of both infiltrating monocytes and neutrophils.

### Resident muscularis macrophages may reduce excessive damage to neuronal and glial networks in WNV-infected mice.

Resident macrophages of the GI tract have been proposed to protect the ENS during bacterial infection by preventing neuronal cell death (12). To address their role after WNV infection, we injected mice with anti-CSF1R mAb, which depletes muscularis macrophages, as confirmed by quantification of Iba1^+^ cells in the myenteric plexus (Supplemental Figure 3, M and N). Animals depleted of muscular macrophages did not show improvement in delayed GI transit times at 7 dpi compared with those given an isotype control mAb (Figure 3J). This GI defect was not caused by a compensatory infiltration of monocytes or monocyte-derived macrophages, as WNV-infected *Ccr2^–/–^* mice treated with anti-CSF1R antibody had similar GI transit delays (Supplemental Figure 3O). However, mice deficient in resident macrophages showed a greater loss of neuronal and glial networks than did isotype control mAb–treated mice after WNV infection (Figure 3, K–M). Thus, resident muscularis macrophages appeared to prevent excessive damage to neuronal and glial networks during WNV infection.

### Damage to neuronal and glial networks is caused by T cells.

**Figure 4:**
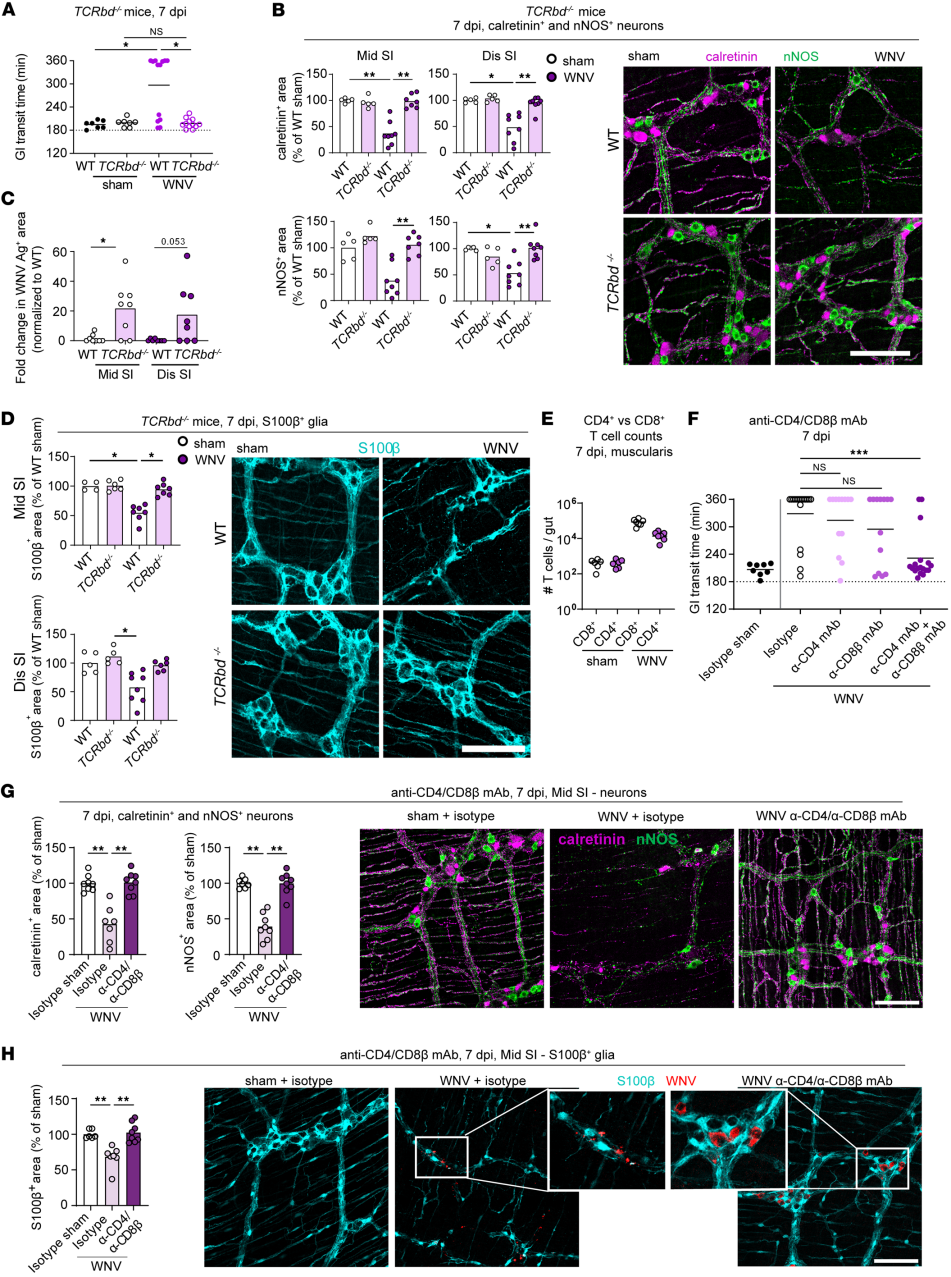
Damage to the neuronal and glial networks is caused by CD4^+^ and CD8^+^ T cells. (**A** and **F**) GI transit was measured after oral gavage of carmine red dye. (**A**) Transit time for sham- or WNV-infected WT or *TCRbd^–/–^* mice at 7 dpi. (**B**–**D**, **G**, and **H**) The muscularis externa was isolated from (**B**–**D**) middle and distal regions of the small intestine of sham- or WNV-infected WT or *TCRbd^–/–^* mice at 7 dpi, (**G** and **H**) middle regions of the small intestine of sham- or WNV-infected WT mice at 7 dpi that were treated with anti-CD4 and/or anti-CD8β or isotype control mAbs and stained for (**B** and **G**) calretinin^+^ and nNOS^+^ neurons, (**C**) WNV antigen, (**D**) S100β^+^ glia, or (**H**) and S100β^+^ glia and WNV antigen. The fraction of the area that stained positive for calretinin, nNOS, or S100β was determined, and the values were normalized to values for (**B** and **D**) WT sham-infected mice or (**G** and **H**) animals treated with an isotype control mAb. Representative images from the myenteric plexus of the middle region of the small intestine. Scale bars: 100 μm. Original magnification, ×2.5 (enlarged insets). (**C**) Data are presented as the percentage of WNV antigen^+^ area in the field of view. (**E**) Counts of live CD45^+^TCRβ^+^CD4^+^ or CD8^+^ T cells in the muscularis of sham- or WNV-infected C57BL6/J mice at 7 dpi. (**F**) Transit time for sham- or WNV-infected mice at 7 dpi; mice were treated with anti-CD4 (α-CD4) and/or anti-CD8β or isotype control mAbs. Data were pooled from (**A**) 3; (**C**–**E**, and **G**) 2; and (**F**) 4 experiments. The numbers of mice per group were as follows: (**A**) *n* = 7–13; (**C** and **D**) *n* = 5–8; (**E**) *n* = 6–7; (**F**) *n* = 8–18; (**G)** *n* = 7–8; (**H**) *n* = 6–8. Lines in **A**, **E**, and **F** and column heights in **B**–**D**, **G**, and **H** indicate mean values. *P < 0.05, **P < 0.01, and ***P < 0.001, by (**A**, **B**, **D**, **G**, and **H**) Kruskal-Wallis ANOVA with Dunn’s post test (all groups compared with each other); (**F**) Kruskal-Wallis ANOVA with Dunn’s post test (compared with the isotype control group); and (**C**) 2-tailed Mann-Whitney *U* test.

We previously noted that CD8^+^ T cells probably contribute to GI tract dysmotility after WNV infection (2). However, these studies were performed with *Cd8*α*^–/–^* mice, which lack CD8^+^ T cells, but retain other T cell subsets (35). Moreover, the effects of WNV infection on the neuronal network in *Cd8*α*^–/–^* mice were not evaluated. To measure GI tract motility and analyze the neuronal and glial networks in the absence of all T cells, *TCRbd^–/–^* mice, which lack both αβ and γδ T cells, were inoculated with WNV. Since mice lacking T cells develop uncontrolled CNS infection and succumb within 10–14 days (36–40), we only performed analyses at 7 dpi. Notably, WNV-infected *TCRbd^–/–^* mice did not show GI tract dysmotility or reduced density of nNOS^+^ and calretinin^+^ neuronal networks in the myenteric plexus as compared with WT littermate controls; infected *TCRbd^–/–^* mice appeared similar to sham-infected WT or *TCRbd^–/–^* mice (Figure 4, A and B), despite the high levels of viral antigen in the myenteric plexus (Figure 4C and Supplemental Figure 4A). Similarly, at 7 dpi, the glial network in the myenteric plexus was unaffected in WNV-infected *TCRbd^–/–^* mice (Figure 4D). In the absence of T cells, the numbers of HuC/D^+^ neurons in the submucosal plexus also were not affected by WNV infection, although the density of the calretinin^+^ network was decreased (Supplemental Figure 4B). These results are consistent with a role for T cells in mediating the injury of motor neurons and glial cells in the myenteric plexus and development of GI tract dysmotility during the acute phase of WNV infection. However, submucosal neurons may be injured by T cell–independent mechanisms. Because *TCRbd^–/–^* mice also have some defects in B cell development and differentiation (41), we performed additional experiments in μMT mice that lack mature B cells and antibody. As WNV-infected μMT and WT littermate mice showed similarly delayed GI transit times at 7 dpi (Supplemental Figure 4C), mature B cells or antibodies were not required for WNV-triggered GI dysmotility.

We next evaluated the specific roles of CD4^+^ and CD8^+^ T cells in WNV-triggered intestinal dysmotility. Flow cytometric analysis and whole-mount staining showed that during the acute phase of WNV infection (days 6 and 7), CD4^+^ and CD8^+^ T cells accumulated in the areas of the muscularis externa adjacent to damaged neurons (Figure 4E and Supplemental Figure 4, D and E). To assess the individual contributions of CD4^+^ and CD8^+^ T cells to WNV-induced GI dysmotility, we treated mice with depleting mAbs that target these T cell subsets (Supplemental Figure 4F). We chose this approach because *Cd4^–/–^* mice also have altered CD8^+^ T cell development due to lineage commitment effects during thymopoiesis (42). Mice treated with anti-CD4 or anti-CD8β antibodies all demonstrated targeted cell depletion in the muscularis externa of the small intestine, the spleen, and Peyer’s patches (Supplemental Figure 4, G–I). Although treatment with either anti-CD4 or anti-CD8β mAbs alone did not rescue intestinal motility at 7 dpi, administration of both anti-CD4 and anti-CD8β mAbs restored intestinal motility to homeostatic levels in most animals (Figure 4F), and this was associated with improved neuronal and glial networks in the myenteric plexus after WNV infection (Figure 4, G and H). These results were similar to those observed in WNV-infected *TCRbd^–/–^* mice, supporting a role for both CD4^+^ and CD8^+^ T cells in the damage of neuronal and glial networks and intestinal dysmotility after WNV infection.

### Antigen-specific CD4^+^ and CD8^+^ T cells cause neuronal and glial injury after WNV infection.

**Figure 5:**
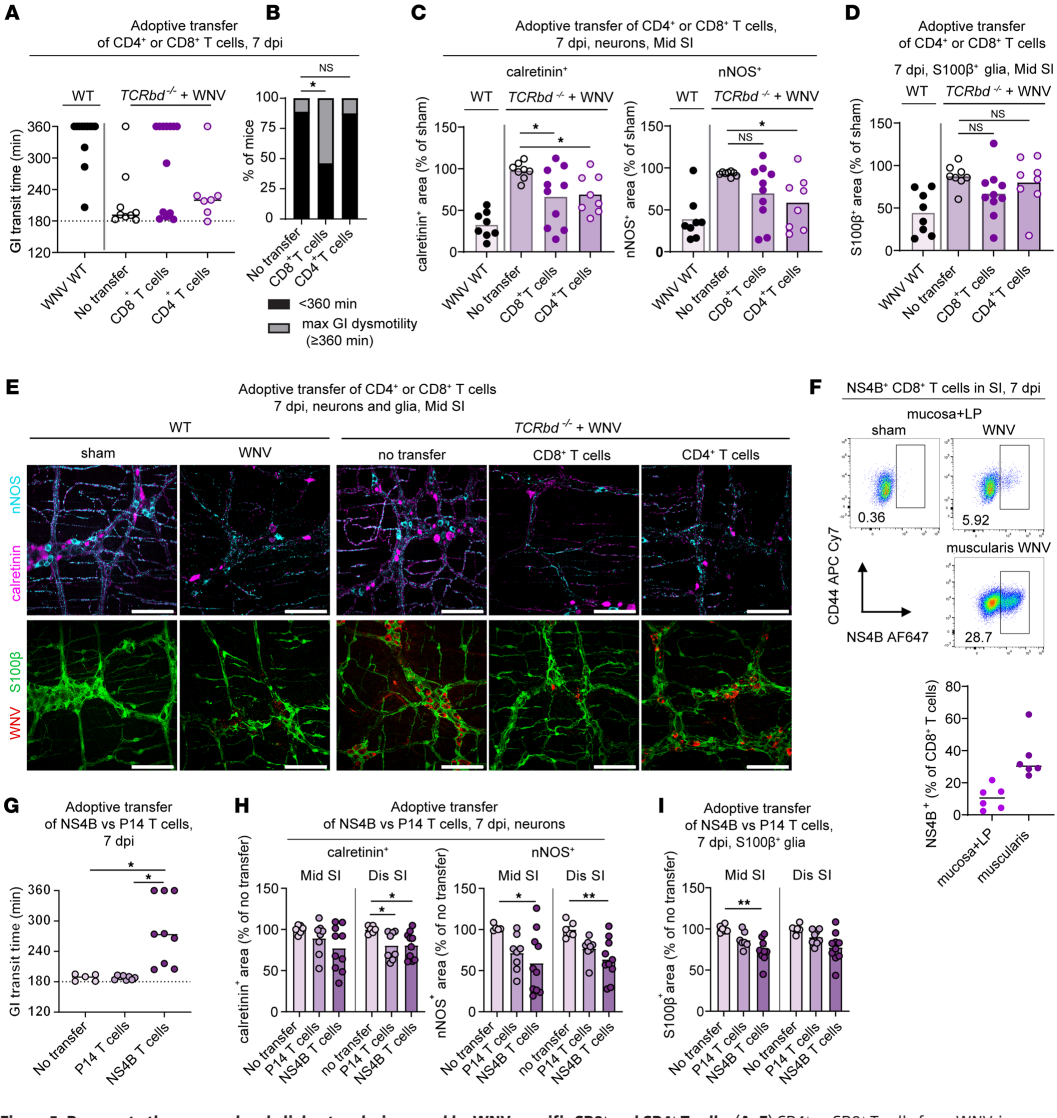
Damage to the neuronal and glial networks is caused by WNV-specific CD8^+^ and CD4^+^ T cells. (**A**–**E**) CD4^+^ or CD8^+^ T cells from WNV-infected WT mice were isolated at 7 dpi and adoptively transferred into *TCRbd^–/–^* mice at 2 dpi. (**A**) GI transit time in recipient *TCRbd^–/–^* mice at 7 dpi, (**B**) proportions of mice with severe GI dysmotility (≥360 min), (**C** and **D**) analysis of neuronal (calretinin, nNOS) and glial (S100β) networks from the middle small intestine at 7 dpi, and (**E**) images obtained from the myenteric plexus of the middle region of small intestine. Scale bars: 100 μm. (**F**) Flow cytometric analysis of the muscularis externa or mucosa and lamina propria at 7 dpi. Cells were stained with mAbs against CD45, TCRβ, TCRγδ, CD8a, CD44, and WNV NS4B D^b^–restricted tetramers and gated on live CD45^+^TCRβ^+^CD8^+^ cells (see Supplemental Figure 4C). Graph shows the percentage of CD8^+^ T cells positive for NS4B. (**G**–**I**) Adoptive transfer of CD8^+^ T cells from P14 transgenic mice (targeting LCMV gp33 peptide) or WNV NS4B transgenic mice into *TCRbd^–/–^* mice. T cells were administered to *TCRbd^–/–^* mice 1 day prior to subcutaneous inoculation with WNV. (**G**) GI transit 7 dpi, (**H** and **I**) analysis of the neuronal (calretinin, nNOS) and glial network (S100β) in middle and distal small intestines at 7 dpi. (**A** and **G**) GI transit was measured after oral gavage of carmine red dye. (**C**, **D**, **H**, and **I**) Fraction of the area that stained positively for calretinin, nNOS, or S100β; values were normalized to (**C** and **D**) WT sham mice or (**H** and **I**) *TCRbd^–/–^* mice without adoptive transfer. Data were pooled from (**A**–**D**) 6; (**F**) 2; and (**G**–**I**) 3 experiments. The numbers of mice per group were as follows: (**F**) *n* = 6; (**A**) *n* = 8–13; (**B**) *n* = 8–13; (**C** and **D**) *n* = 8–13; (**F**) *n* = 6; (**G**) *n* = 5–9; (**H** and **I**) *n* = 6–10. Lines in **G** and **F** and column heights in **C**, **D**, **H**, and **I** indicate mean values. *P < 0.05 and **P < 0.01, by (**C** and **D**) ANOVA with Dunnett’s post test (comparison with the no-transfer group); (**B**) χ^2^ test with Bonferroni correction (proportions compared with the no-transfer group); and (**G**–**I**) Kruskal-Wallis ANOVA with Dunn’s post test (comparison with the no-transfer group).

To further dissect the specific contribution of CD4^+^ and CD8^+^ T cells in WNV-infected mice to neuronal and glial damage, we isolated WNV-primed CD4^+^ and CD8^+^ T cells from WT mice at 7 dpi and adoptively transferred them into WNV-infected *TCRbd^–/–^* mice at 2 dpi (Supplemental Figure 5A). Subsequently, we analyzed intestinal tract motility and injury to neurons and glia at 7 dpi (Figure 5, A–E) after confirming the efficiency of the T cell transfers (Supplemental Figure 5, B and C). More than 50% of *TCRbd^–/–^* mice that received CD8^+^ T cells developed severe GI tract dysmotility (≥360 minutes) and showed damage to neuronal and glial networks, whereas sham or WNV-infected *TCRbd^–/–^* mice without T cell transfers did not (Figure 5, A–E). Although most WNV-infected mice injected with CD4^+^ T cells did not show delayed GI tract transit times, we still observed injury to neurons (Figure 5, A–E). Together, these results suggest that, while both CD4^+^ and CD8^+^ T cells could injure the neuronal network, CD8^+^ T cells triggered greater damage resulting in intestinal dysmotility.

Virus-specific and bystander effector CD8^+^ T cells can target infected cells for lysis and promote local inflammation (43). To determine the role of antigen-specific recognition of target cells by CD8^+^ T cells in ENS injury, we stained cells with D^b^-restricted tetramers that recognize an immunodominant peptide epitope (SSVWNATTAI) in the WNV NS4B protein (44). In WNV-infected small intestines, approximately 25% of CD8^+^ T cells in the muscularis externa (and 10% in the remainder of the small intestine) were specific for the NS4B immunodominant peptide (Figure 5F). To determine the contributions of antigen-specific and bystander CD8^+^ T cells to the damaging of neurons and glia after WNV infection, we utilized T cell receptor–transgenic (TCR-transgenic) mice in which the vast majority of CD8^+^ T cells were specific for the WNV NS4B peptide epitope or, as a control, for the lymphocytic choriomeningitis virus (LCMV) gp33 peptide epitope (KAVYNFATC) (45–47). Transgenic WNV NS4B or LCMV gp33 CD8^+^ T cells were adoptively transferred into *TCRbd^–/–^* mice, and, 1 day later, recipient animals were inoculated subcutaneously with WNV (Supplemental Figure 5, D and E). At 7 dpi, we measured intestinal tract motility, collected mesenteric lymph nodes to confirm T cell colonization (Supplemental Figure 5F), and analyzed the neuronal and glial networks from the middle and distal regions of the small intestine. Whereas *TCRbd^–/–^* mice given NS4B-specific CD8^+^ T cells showed delayed intestinal transit times and damage to neuronal and glial networks in the middle and distal regions of the small intestine, animals given LCMV gp33–specific (P14) CD8^+^ T cells did not develop dysmotility (Figure 5, G–I). Nonetheless, WNV-infected *TCRbd^–/–^* mice that received LCMV-specific CD8^+^ T cells showed some damage to neuronal networks (Figure 5H), suggesting either limited bystander injury mediated by antigen-nonspecific CD8^+^ T cells or expansion of WNV-specific CD8^+^ T cells from the small repertoire of endogenous TCRs in the LCMV gp33 (P14) TCR-transgenic mice on a WT C57BL/6 background.

To further assess the potential role of bystander CD8^+^ T cells, we crossed the P14 LCMV-transgenic mice with *Rag1^–/–^* mice to generate animals in which virtually every CD8^+^ T cell was specific for the LCMV gp33 peptide (Supplemental Figure 5G); these animals also lacked CD4^+^ T cells. We measured the intestinal transit in WT and *P14 Rag1^–/–^* mice 7 days before (baseline) and after WNV infection (Supplemental Figure 5, G–I). Notably, WNV-infected *P14 Rag1^–/–^* mice showed normal GI tract transit at day 7, comparable to baseline and sham-infected WT mice (Supplemental Figure 5H). Similarly, we did not observe damage to neuronal or glial networks in WNV-infected *P14 Rag1^–/–^* mice (Supplemental Figure 5I). Collectively, these results did not indicate a substantive role for bystander CD8^+^ T cells in neuronal or glial injury in the context of WNV infection.

We similarly assessed the role of bystander CD4^+^ T cells on ENS integrity and function after WNV infection, by crossing OT-II TCR transgenic mice with *Rag1^–/–^* mice to generate mice that lack CD8^+^ T cells and have CD4^+^ T cells that are specific for the OVA peptide 323-339 (ISQAVHAAHAEINEAGR) (Supplemental Figure 5J). At 7 dpi, none of the WNV-infected *OTII Rag1^–/–^* mice exhibited intestinal dysmotility, and neuronal networks appeared normal (Supplemental Figure 5, K and L). Thus, bystander CD4^+^ T cells also did not induce neuronal injury and GI dysmotility after WNV infection.

### CD4^+^ and CD8^+^ T cells cause neuron and glia injury using multiple effector mechanisms.

**Figure 6:**
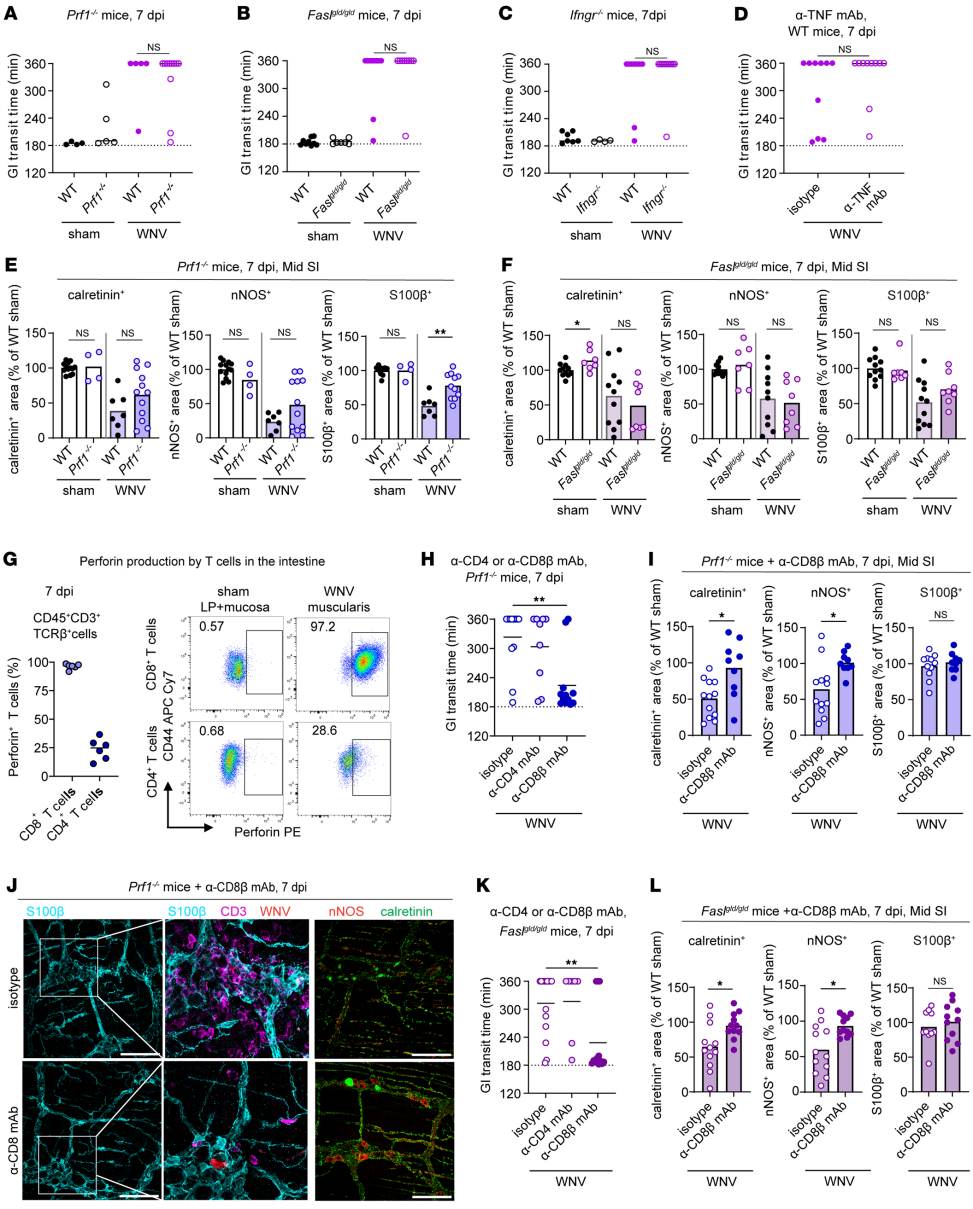
CD4^+^ and CD8^+^ T cells injure neurons and glia using multiple effector functions. (**A**–**D**, **H**, and **K**) GI tract transit was measured after oral gavage of carmine red dye at 7 dpi. Transit time for sham, WNV-infected WT, or WNV-infected (**A**) *Prf1^–/–^*, (**B**) *Fasl^gld/gld^*, (**C**) *Ifngr^–/–^*, and (**D**) WT mice treated with anti-TNF or isotype control mAb or WNV-infected (**H**) *Prf1^–/–^* or (**K**) *Fasl^gld/gld^* mice treated with anti-CD4, anti-CD8β, or isotype control mAb. (**E**, **F**, **I**, **J**, and **L**) The muscularis externa was isolated from the middle regions of small intestines from sham-infected, WNV-infected WT, or *Prf1^–/–^* mice (**E**), *Fasl^gld/gld^* mice (**F**), WNV-infected *Prf1^–/–^* mice (**I** and **J**), or *Fasl^gld/gld^* mice (**L**) treated with anti-CD4 or anti-CD8β mAb at 7 dpi and then stained. The fraction of the area that stained positive for calretinin, nNOS, or S100β was determined, and values were normalized to those for sham-infected WT mice. (**J**) Representative images were obtained from the myenteric plexus of the middle region of the small intestine. Scale bars: 100 μm. Original magnification, ×2.5 (enlarged insets). Data were pooled from (**A**–**C**, **F**, **I**, and **J**) 3; (**D**, **G**, and **L**) 2; (**E** and **K**) 5; and (**H**) 4 experiments. The numbers of mice per group were as follows: (**A**) *n* = 4–11; (**B**) *n* = 7–10; (**C**) *n* = 4–11; (**D**) *n* = 10; (**E**) *n* = 4–13; (**F**) *n* = 6–11; (**G**) *n* = 6; (**H**) *n* = 9–12; (**I**) *n* = 9–12; (**K**) *n* = 7–15; (**L**) *n* = 6–7. Lines indicate (**A**–**D** and **G**) median or (**H** and **K**) mean values, and column heights indicate the mean values. *P < 0.05 and **P < 0.01, by (**A**–**F**, **I**, and **L**) Mann-Whitney *U* test and (**H** and **K**) ANOVA with Dunnett’s post test (comparison with the isotype control group).

T cells can use a variety of mechanisms to clear WNV-infected neurons from the brain including cytotoxic granules (perforin/granzymes), proinflammatory cytokines (e.g., TNF and IFN-γ), and death receptor signaling pathways (Fas ligand [FasL] or TRAIL) (37, 48–52). To determine the contribution of these T cell effector mechanisms to ENS damage after WNV infection, we used a combination of genetic and pharmacological loss-of-function approaches to test the role of perforin (*Prf1^–/–^* mice), FasL (*gld*-mutant; *Fasl^gld/gld^* mice), IFN-γ (*Ifngr^–/–^* mice), and TNF (blocking mAb, MP6-XT22 that inhibits both membrane-associated and soluble forms; ref. 53). At 7 dpi, WNV-infected *Prf1^–/–^*, *Fasl^gld/gld^*, and *Ifngr^–/–^* mice all showed delayed GI transit like the WT littermate controls (Figure 6, A–C). WT mice treated with an anti-TNF mAb (MP6-XT22) also showed delayed GI transit times after WNV infection similar to controls (Figure 6D and Supplemental Figure 6A). In addition, we observed similar or even greater intestinal segment dilation in WNV-infected *Prf1^–/–^*, *Fasl^gld/gld^*, *Ifngr^–/–^*, and anti-TNF–treated mice compared with WNV-infected control mice (Supplemental Figure 6B). Although we observed damage to the neuronal networks and high numbers of CD3^+^ T cells in the myenteric plexus in WNV-infected *Prf1^–/–^* and *Fasl^gld/gld^* mice, the glial network appeared more intact in these 2 strains (Figure 6, E and F, and Supplemental Figure 6, C and D). These data suggest that loss of individual cytolytic pathways or effector cytokines was not sufficient to ameliorate the WNV-induced damage of neurons and intestinal dysmotility, although glia could be affected.

Activated CD4^+^ and CD8^+^ T cells can both have cytolytic activity (54) and produce inflammatory cytokines after WNV antigen stimulation (37, 55). Flow cytometric analysis revealed that perforin was present in almost all CD8^+^ T cells and, on average, 25% of CD4^+^ T cells in the muscularis layer of WNV-infected mice at 7 dpi (Figure 6G). As multiple effector functions in different T cell populations could be used concurrently to target WNV-infected cells, we hypothesized possible redundancy in effector mechanisms that cause injury to enteric neurons. To test this hypothesis, prior to WNV infection, we depleted CD4^+^ or CD8^+^ T cells in either *Prf1^–/–^*, *Fasl*^gld/gld^, or *Ifngr^–/–^* mice or in WT mice treated with blocking mAbs against IFN-γ (Supplemental Figure 6A). Depletion of CD4^+^ or CD8^+^ T cells did not mitigate GI transit defects in either WNV-infected *Ifngr^–/–^* or WT mice treated with an IFN-γ–blocking mAb (Supplemental Figure 6, E and F). However, depletion of CD8^+^, but not CD4^+^, T cells in *Prf1^–/–^* and *Fasl^gld/gld^* mice normalized the GI transit time defect and prevented damage to neuronal networks (Figure 6, H–L, and Supplemental Figure 6G). These data suggest that (a) CD8^+^ T cells can utilize an alternative cytolytic mechanism in the absence of perforin and (b) CD4^+^ T cells require both perforin and FasL pathways to mediate WNV-induced damage and dysmotility in the context of a CD8^+^ T cell deficiency. Thus, T cells can use multiple effector mechanisms to target WNV-infected neuronal cells in the small intestine.

### Mice lacking both perforin and FasL do not develop GI dysmotility and have intact neuronal and glial networks.

**Figure 7:**
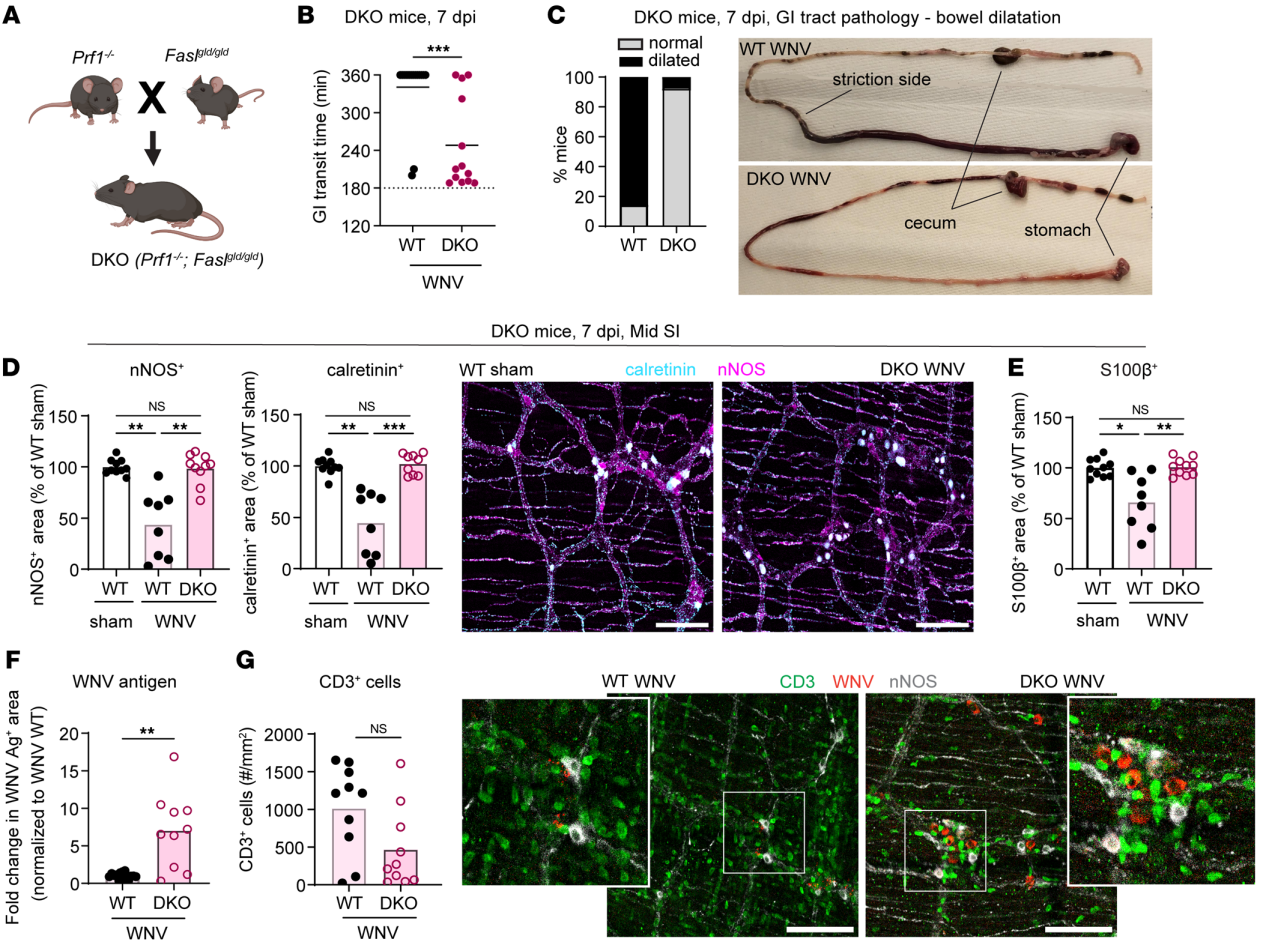
Mice lacking perforin and Fas/FasL signaling do not develop WNV-triggered GI dysmotility or neuronal and glial network injury. (**A**) Scheme for the generation of *Prf1^–/–^ Fasl^gld/gld^* (DKO) mice. The figure in **A** was created using BioRender software. (**B**) GI transit time in WNV-infected WT or DKO mice at 7 dpi. (**C**) Proportions of WNV-infected WT and DKO mice showing abnormal bowel dilation in the small intestine at 7 dpi. (**D**–**G**) The muscularis externa was isolated from the middle regions of small intestines from sham- (**D** and **E**) or WNV-infected WT or DKO (**F** and **G**) mice at 7 dpi and stained. (**D**–**F**) The fraction of the area that stained positive for calretinin, nNOS, S100β, and WNV antigen was determined, and values were normalized to those for (**D** and **E**) sham-infected WT mice or (**F**) WNV-infected WT mice. (**G**) The numbers of CD3^+^ cells in the myenteric plexus were calculated by dividing the area positive for CD3 staining with the average size of CD3^+^ cells. Cell counts are expressed as the number of CD3^+^ cells per mm^2^. (**D** and **G**) Representative images of the myenteric plexus of the middle region of the small intestine. Scale bars: 100 μm. Original magnification, ×2.5 (enlarged insets). Data were pooled from (**B**, **C**, and **F**) 5 and (**D**, **E**, and **G**) 4 experiments. The numbers of mice per group were as follows: (**B** and **C**) *n* = 13–16; (**D** and **E**) *n* = 8–10; (**F**) *n* = 10–15; (**G**) *n* = 10. Lines in **B** and column heights in **D**–**G** indicate mean values. *P < 0.05, **P < 0.01, and ***P < 0.001, by (**B**, **F**, and **G**) 2-tailed Mann-Whitney *U* test and (**D** and **E**) Kruskal-Wallis ANOVA with Dunn’s post test.

FasL and perforin can function together to augment cytotoxic T cell responses (56). To more definitively determine whether perforin and FasL together are the dominant mechanisms causing neuronal and glial injury and ensuing GI dysmotility after WNV infection, we generated double-KO (DKO) mice by crossing *Prf1^–/–^* and *Fasl^gld/gld^* mice (Figure 7A). We inoculated DKO (*Prf1^–/–^* *Fasl^gld/gld^*) subcutaneously with WNV and at 7 dpi measured the GI transit time and analyzed neuronal and glial networks in the small intestine. Most WNV-infected DKO mice showed normal GI motility and an absence of bowel dilation (Figure 7, B and C). Consistent with these results, the neuronal and glial networks were intact in WNV-infected DKO mice and like those of uninfected WT mice (Figure 7, D and E), despite the presence of WNV antigen and CD3^+^ T cells in the myenteric plexus of DKO mice (Figure 7, F and G). These results indicate that in mice containing both CD4^+^ and CD8^+^ T cells, either FasL or perforin was sufficient to cause the WNV-triggered pathology in the gut. When both effector mechanisms or T cells were absent, WNV-induced defects in GI motility and neuronal and glial network injury were prevented.

## Discussion

In this study, we show that WNV infection damaged enteric neuronal and glial networks, and this injury was associated with reduced intestinal motility. Acute WNV infection causes loss of neuronal bodies in both the myenteric and submucosal plexuses and diminished density of neuronal fibers in the circular muscle layer. We observed a durable loss of neurons in the middle region of the small intestine, which is associated with a lower density in the networks of major neuronal subgroups (calretinin^+^ and nNOS^+^) and 5-HT^+^ interneurons. These neuronal defects may be perpetuated by the presence of persistent WNV RNA in the intestine (2). Persistent viral RNA can be detected directly or indirectly by immune cells, which can produce proinflammatory mediators or recruit cells that contribute to the continued loss of neuronal networks and hinder recovery. Despite an accumulation of monocytes and macrophages at the site of neuronal infection in the proximity of the myenteric plexus, infection-induced injury to neurons and glia is caused principally by WNV-specific CD8^+^ T cells with a contribution from CD4^+^ T cells. Furthermore, the T cell–mediated injury of ENS components is mediated by a combination of multiple redundant effector functions, including Fas-FasL signaling and perforin, that contribute to the damage of neurons and glia. When both effector mechanisms are absent, the intestinal dysmotility and damage to neuronal and glial networks following WNV infection are prevented.

The damage to the enteric glia by WNV is important, as these cells express receptors for neurotransmitters that sustain neuronal circuits and regulate GI motility (4, 20, 57, 58). To date, no studies have shown the extent of enteric glial damage during viral or bacterial infection. Despite being infected by WNV with less frequency than neurons, glial networks and processes were diminished during the acute phase of WNV infection. Although direct infection could have a role in glial injury, the inflammatory environment or actions of infiltrating immune cells also may contribute to their damage. A loss of S100β^+^ glial cells or glial networks was linked previously to increased proinflammatory cytokine levels (e.g., IFN-γ and TNF-α) in patients with inflammatory bowel disease (59). Our data suggest that T cells used cytolytic mechanisms (perforin or Fas/FasL signaling) to induce glial injury in the context of WNV infection. The damage to both neurons and glia during the acute phase of infection likely exacerbated the GI dysmotility phenotype. The persistent reduction of the glial network, especially in the middle (jejunal) region of the small intestine, also likely adversely affected recovery of the ENS, as glia can serve as a source of new neurons via differentiation after injury and produce glia cell–derived neurotrophic factor (GDNF) and nerve growth factor (NGF) (24, 60–62).

The dysmotility associated with relative loss of specific neuronal subpopulations was reported in response to bacterial intestinal infection (7, 12), although in contrast to these studies, we did not observe differences in the ratios of the major nNOS^+^ and calretinin^+^ subpopulations after WNV infection. Instead, WNV triggered losses in the axonal network of serotonergic neurons. While we did not observe a decrease in the axonal network of serotonergic neurons during the acute phase of WNV infection, we detected durable losses of the serotonergic neurons in the myenteric plexus at 14 dpi and through 65 dpi. Although their precise role in GI motility has been debated (63), the disruption of serotonergic neuronal signaling after WNV infection might delay the replenishment and repair of neuronal networks through effects on neurogenesis (18).

In the context of tissue inflammation, infiltrating monocytes can differentiate into macrophages and produce proinflammatory mediators including TNF, IL-6, IL-1β, and ROS. After HSV-1 infection or in an experimental model of inflammatory bowel disease, Ly6C^hi^CCR2^+^ monocytes and derived macrophages contributed to the damage to enteric neurons (14, 27, 64, 65). Although CCR2^+^ monocytes and newly differentiated macrophages (Iba1^+^CCR2^+^) localized near WNV-infected neurons at 6 dpi and persist through 15 dpi, acquired or genetic depletion of monocytes did not prevent the neuronal damage and the ensuing WNV-induced GI tract dysmotility. Thus, WNV-triggered dysmotility during the acute phase appeared to be independent of the actions of infiltrating monocytes and monocyte-derived macrophages. Moreover, resident muscularis macrophages also did not contribute to the ENS damage. Instead, depletion studies showed that resident muscularis macrophages limited neuronal death and glial injury in WNV-infected intestines, which supports findings showing their protective effects in the context of some bacterial infections (12, 66). Although the mechanistic link to protection by muscularis macrophages remains unclear, we noted higher levels of *Ccl6* in our neuron-enriched RNA-Seq analysis, which is thought to polarize macrophages toward a prohealing phenotype (67).

Our prior study suggested that T cells in the GI tract contribute to motility defects after WNV infection (2). We extended these results using antibody depletions, genetically deficient mice, and adoptive transfer experiments, which together established contributory and pathogenic roles for both CD4^+^ and CD8^+^ T cells. Here, we show that *TCRbd^–/–^* mice lacking both CD4^+^ and CD8^+^ T cells had intact neuronal networks in the myenteric but not the submucosal plexus. This discrepancy might have been due to the position of the submucosal neurons closer to the lamina propria, where other innate immune cells were located and could contribute to neuronal damage. Transfer of CD8^+^ T cells from WNV-primed WT mice or naive WNV NS4B peptide TCR transgenic mice into recipient *TCRbd^–/–^* mice resulted in dysmotility and injury to the neuronal networks in the context of WNV infection. However, experiments with OT-II or P14 LCMV transgenic mice suggested that the damage was principally mediated by antigen-specific and not bystander T cells. Even though bystander T cells can mediate protective or pathogenic roles in the context of some viral infections (68, 69), we did not observe substantive contributions by these cells to ENS injury.

Whereas adoptive transfer of WNV-primed WT CD8^+^ T cells to WNV-infected *TCRbd^–/–^* mice was sufficient to induce GI dysmotility in more than 60% of mice, few mice showed severe ENS damage, and the glial network density was not significantly decreased. Moreover, adoptive transfer of CD4^+^ T cells from WNV-infected WT mice to WNV-infected *TCRbd^–/–^* mice triggered low levels of neuronal damage, which was not sufficient to cause severe GI dysmotility. These partial phenotypes were consistent with our observation that in WT mice, depletion of both CD4^+^ and CD8^+^ T cell populations was required to prevent GI tract injury and dysmotility after WNV infection. While WNV-specific CD8^+^ T cells appeared to be the dominant mediators of neuronal damage and intestinal transit dysfunction, there were clearly contributory pathological effects of CD4^+^ T cells, which were capable of inducing dysmotility in WNV-infected mice when CD8^+^ T cells were depleted. Redundant pathogenic roles of CD4^+^ and CD8^+^ T cells were described for mouse hepatitis virus (MHV) in the context of demyelination in the brain (70, 71). During MHV infection, IFN-γ produced by CD8^+^ T cells was the primary agent of demyelination, whereas IFN-γ was not essential for GI dysmotility in WNV-infected mice. Similarly, and despite being implicated in the damage to enteric neurons in a model of ganglionitis (72), TNF was not required for intestinal motility defects in WNV-infected mice. Instead, our T cell depletion studies in KO mice suggest that both perforin and FasL-dependent cytolytic mechanisms contributed to neuronal and glial injury in the GI tract of WNV-infected mice. Our experiments in DKO mice showed that both perforin and FasL-dependent mechanisms contributed to the pathologic changes in the small intestine during WNV infection. As with *TCRbd^–/–^* mice, we also observed higher levels of WNV Ag in neurons at 7 dpi in the DKO mice. This result, together with our observation of intact neuronal and glial networks, confirms the important role of FasL and perforin as the principal damaging T cell effector mechanisms during the immune response to WNV in the gut.

We acknowledge several limitations of our studies. (a) We used WNV as a model of infection to study acute and chronic GI dysmotility of the small intestine, even though a similar syndrome has not been definitively demonstrated in humans. (b) For studies with *TCRbd^–/–^* mice or animals lacking T cells due to depletion with antibodies, we were limited to evaluating early time points for GI tract motility measurements because these mice succumb to uncontrolled WNV infection in the brain and spinal cord at later time points. (c) Since all neurons in the brain and spinal cord are susceptible to WNV infection, we cannot rule out an effect of CNS dysfunction on the gut motility phenotype. Measurements of peristalsis and GI tract function in isolated intestines from WNV-infected mice might address this problem. (d) Some treatments (e.g., anti-CSF1R antibody) led to dysmotility that lasted more than 6 hours; however, we were not able to quantify this effect further due to time limitations in our mouse facility. (e) Our experiments were performed with male and female mice that were randomly assigned to specific experimental groups. While some studies show differences in GI tract motility depending on the sex of the mice or the phase of the estrous cycle (73–75), we observed similar phenotypes in male and female mice. However, we did not specifically test for effects of WNV infection on GI tract motility in females at proestrus, estrus, metestrus, or diestrus phases. (f) We observed an imperfect correlation between damage to neurons and glia and GI dysmotility, which might be due to tissue sampling bias. Functional experiments ex vivo that stimulate neurons from WNV-infected mice might provide more precise correlations.

In summary, our experiments show how the effector functions used by infiltrating antigen-specific CD4^+^ and CD8^+^ T cells can rapidly injure the neurons and the neighboring glia, resulting in durable tissue damage and long-term intestinal transit dysfunction. Pharmacological control of these T cell effector functions may be challenging, given the need to prevent sustained neurotropic viral infection that intrinsically can cause damage in the GI tract and other tissues, like the brain and spinal cord.

## Methods

### Sex as a biological variable.

Our study examined male and female animals, and similar findings are reported for both sexes.

### Mice.

WT C57BL/6J (no. 000664), *TCRbd^–/–^* (B6.129P2-Tcrbtm1Mom/J; no. 002122), *Prf1^–/–^* (C57BL/6-Prf1tm1Sdz/J; no. 002407), *FasL^gld/gld^* (B6Smn.C3-Faslgld/J, no. 001021), *Ifngr^–/–^* (B6.129S7-Ifngr1tm1Agt/J; no. 003288), *ChaT* GFP [B6.Cg-Tg(RP23-268L19-EGFP)2Mik/J; no. 007902], *Ccr2 GFP* [B6(C)-Ccr2tm1.1Cln/J; no. 027619], Ai3 [B6.Cg-Gt(ROSA)26Sortm3(CAG-EYFP)Hze/J; no. 007903], and *Rag1^–/–^* (B6.129S7-*Rag1^tm1Mom^*/J, no. 002216; P14 LCMV TCR [B6;D2-Tg(TcrLCMV)327Sdz/JDvsJ], no. 004694; OTII [B6.Cg-Tg(TcraTcrb)425Cbn/J], no. 004194) mice were obtained commercially from The Jackson Laboratory. *Snap25l10a* GFP mice [B6;FVB-Tg (Snap25-EGFP/Rpl10a)JD362Htz/J; no. 030273; The Jackson Laboratory] were provided by Joseph D. Dougherty (Washington University in St. Louis, St. Louis, Missouri, USA). *Ccr2* CreER mice were provided by Burkhard Becher (University of Zurich, Zurich, Switzerland) and crossed with *Ai3* (Rosa26 YFP) mice to obtain *Ccr2* CreER *Rosa26 YFP* mice. *DKO* (*Prf1^–/–^ Fasl*^gld/gld)^ mice were generated by crossing *Prf^–/–^* mice with *Fasl^gld/gld^* mice. Heterozygous *Prf1^+/–^ Fasl^gld/+^ mice* were mated with *Prf1^–/–^*, and *Prf1^–/–^ Fasl^gld/+^* mice were selected for brother-sister matings to obtain DKO mice. All mice were bred under pathogen-free conditions at Washington University School of Medicine.

Nine- to 10-week-old male or female C57BL6/J mice or transgenic and KO mice were inoculated with 10^2^ focus-forming units (FFU) of WNV in 50 μL PBS via subcutaneous injection into the footpad. All dissections and inoculations were performed under anesthesia, induced and maintained by using ketamine hydrochloride and xylazine or isoflurane, and every effort was made to minimize suffering.

### Viruses.

WNV New York 1999, clone 382-99 (76), was propagated in Vero cells (passage 1) as described previously (2). Virus stocks were titrated using a focus-forming assay on Vero WHO cells (2).

### GI tract motility measurements.

GI tract motility was assessed as described previously (77). Briefly, mice were administered 300 μL 6% (w/v) carmine red dye (MilliporeSigma) in 0.5% methylcellulose diluted in sterile water by oral gavage. After 3 hours, mice were placed individually into cardboard boxes, and fecal pellets were examined for red color every 5–10 minutes.

### Enrichment and adoptive transfer of T cells.

Spleens and mesenteric lymph nodes from isoflurane-overdosed, WNV-infected (7 dpi) WT or naive WNV NS4B and LCMV P14 TCR transgenic mice were harvested, and single-cell suspensions were obtained by mashing with a syringe plunger through a cell strainer (70 μm), followed by lysis of erythrocytes using ACK lysis buffer for 3 minutes on ice. After washing with PBS plus 0.5% BSA and 2 mM EDTA, cells were counted, and the single-cell suspension was enriched for CD4^+^ or CD8^+^ T cells by negative selection using a CD4 or CD8a T cell Isolation Kit (Miltenyi Biotec; 130-104-454 and 130-104-075) following the manufacturer’s instructions. For the transfer of WNV NS4B and P14 TCR T cells, *TCRbd^–/–^* mice were administered 10^6^ CD8^+^ T cells in 100 μL PBS via retro-orbital injection 1 day prior to WNV infection. CD4^+^ (10^7^ cells) or CD8^+^ (5 × 10^6^ cells) T cells were administered via retro-orbital injection into *TCRbd^–/–^* mice 2 days after WNV infection. For each experiment, the efficiency of T cell enrichment and transfer was assessed by flow cytometry.

Additional details on materials and methods are provided in the Supplemental Methods.

### Statistics.

We performed statistical analyses using GraphPad Prism 9.0 (GraphPad Software). A 2-tailed Mann-Whitney *U* test, 1-way Kruskal-Wallis ANOVA with Dunn’s correction, 1-way ANOVA with Dunnett’s correction, and χ^2^ test with Bonferroni correction were used to determine significance depending on the number of comparison groups and the data variance. A *P* value of less than 0.5 was considered statistically significant. Data indicate the mean ± SEM.

### Study approval.

This study was conducted in accordance with the recommendations of the NIH’s *Guide for the Care and Use of Laboratory Animals* (National Academies Press, 2011). Animal experiments were performed as specified in protocols approved by the IACUC of the Washington University School of Medicine (assurance number A3381-01).

### Data availability.

Data are available from the corresponding author upon request. All data supporting the graphs are provided in the Supporting Data Values file. RNA-Seq data generated in this study have been deposited in the NCBI’s Gene Expression Omnibus (GEO) database (GEO GSE264415).

## Author contributions

HJ performed all mouse treatments and infections, tissue harvesting, flow cytometry, confocal imaging, gut motility assays and analysis. FRZ and PD performed tissue harvesting, flow cytometry, and RNA-Seq analysis and helped with the experimental design. MM provided the anti-CCR2 mAb. HJ, LBT, TSS, and MSD designed the experimental studies and analyzed results. HJ, TSS, and MSD wrote the initial draft of the manuscript, with all other authors providing editorial comments.

## Supplementary Material

Supplemental data

Supporting data values

## Version 1 08/29/2024

In-Press Preview

## Version 2 11/01/2024

Electronic publication
