# Supplementary material for: West Nile virus triggers intestinal dysmotility via T cell–mediated enteric nervous system injury
Source: J Clin Invest. 2024 Aug 29;134(21):e181421. doi: 10.1172/JCI181421 (PMC11527448; doi:10.1172/JCI181421)
Supplement: Supplemental data [file jci-134-181421-s153.pdf]

Supplemental Material

West Nile virus triggers intestinal dysmotility via T cell-mediated enteric nervous system injury

Hana Janova[1], Fang R. Zhao[1], Pritesh Desai[1], Matthias Mack[2], Larissa B. Thackray[1], Thaddeus S. Stappenbeck[3]*, and Michael S. Diamond[1,4,5,6]*

Materials and Methods

Immunofluorescence microscopy. Intestines were harvested from isoflurane overdosed mice at indicated time points after WNV inoculation. To examine the middle region of the small intestine, 5 cm from the same anatomical regions were used. Distal small intestine regions were defined as last 5 cm preceding the cecum, and the proximal part of small intestine was defined as the first 2 cm of small intestine. The intestinal contents were flushed out sequentially with PBS and 4% paraformaldehyde (PFA). The tissue was opened along the mesenteric border, uniformly stretched, pinned to Sylgard silicon plates, and fixed in 4% PFA overnight at 4°C. The fixed tissue was washed three time in PBS and unless processed at the same day, stored in 0.02% NaN_3 in PBS. For whole mount staining of myenteric plexus, the mucosa and muscularis mucosa together with the attached submucosal plexus were dissected away from the muscularis externa. To analyze the submucosal plexus, the mucosa was scratched and muscularis mucosae was carefully dissected leaving the submucosal layer intact. All dissections were performed using a dissecting microscope (Olympus SZX7). The dissected tissue was blocked for at least 1 h at room temperature (RT) in blocking buffer (5% normal donkey serum (NDS) + 1% bovine serum albumin (BSA) + 1% Triton X-100 in 1x PBS or 1x Tris buffered saline (TBS) (WNV antigen staining) and incubated overnight in 3% NDS + 1% BSA + 1% Triton X-100 in 1x PBS with primary antibodies

against nNOS (goat, 1:500, Abcam; # Ab1376), nNOS (goat, 1:500, Millipore; # AF2416), calretinin clone DC8 (rabbit, 1:500, ThermoFisher Scientific, # 180211), Iba1 (rabbit, 1:500, Abcam; # ab178846), HuC/D (biotinylated mouse, 1:150, ThermoFisher Scientific, # A21272), Tuj1 (rabbit, 1:500, Abcam, # ab18207), serotonin (goat, 1:500, Immunostar, # 20079), S100β clone EP1576Y (rabbit, 1:500, 1:500, Abcam, , # ab52642), cKit (goat, 1:200, R&D, # AF1356), CD3 (rabbit, 1:150, Abcam, # ab5690), CD3 (Armenian hamster, 1:100, eBioscience, # 13-0031-8), CD4 (rat, 1:150, eBioscience, # 14-0041-82), CD8b APC (rat, 1:150, BioLegend, # 126613), peripherin (rabbit, 1:1,000, Millipore, # AB1530), or in 3% NDS + 1% BSA + 1% Triton X-100 in TBS with rat anti-WNV polyclonal serum (1:500) (1) at 4°C. For HuC/D staining, tissue was pre-treated with Avidin/Biotin Blocking Kit (Vector Laboratories; # SP-2001) following the manufacturer instructions. Tissue was washed three times with PBS + 1% Triton X-100 and incubated for 1 h at room temperature with secondary antibodies: donkey anti-rabbit Alexa Fluor 488, 647, 546 (1:500, ThermoFisher Scientific, # A31573, A21206, A10040) or DyLight 405 (1:250, Jackson ImmunoResearch laboratories, # 711-475-152), donkey anti-goat Alexa Fluor 488, 647, 546 (1:500, ThermoFisher Scientific, # A11055, A32849, A11056) and donkey anti-rat Alexa Fluor 555, 488, 647 (1:500, ThermoFisher Scientific, # A78945, A21208, S78947) or Streptavidin Alexa Fluor 546 (1:500, ThermoFisher Scientific, # S11225) in PBS or TBS + 3% NDS + 1% BSA +1% Triton X-100. Subsequently, tissue sections were washed three times in PBS + 1% Triton X-100, counterstained for nuclei with Hoechst 33342 dye in PBS (1:5,000, ThermoFisher Scientific) and mounted in Aqua-Poly/Mount (Polysciences) or ProLong™ Glass Antifade Mountant (ThermoFisher Scientific, # P36980).

Images were captured on a Zeiss LSM880 Laser Scanning Confocal microscope using 20x (NA 0.8) or 40x (NA 1.4) objective. The scanned images were processed and analyzed by Fiji software (https://fiji.sc/Fiji). To quantify the density of neuronal (peripherin, nNOS, calretinin, 5-HT), glial (S100β) and ICCs (cKit) networks, four to five random regions (0.65 mm^2) were scanned using 20x (NA 0.8) objective and stitched (ZEN black software, Zeiss). To assess the level of WNV

infection, 4 mm^2 regions of tissue were scanned and stitched (ZEN black software, Zeiss). For all images, the background signal was uniformly subtracted (rolling ball, radius 50). Quantification was performed by converting images to binary using a threshold tool, and a fraction of total area was measured. For cell density analysis, the median value from each 4-5 images was calculated for every mouse and normalized to values of respective controls (as described in the legends).

The fraction of total area with WNV-antigen staining in *TCRbd*$^{-/-}$ *and DKO (Prf1*$^{-/-}$*; FasL*$^{gld/gld}$*)* mice was expressed as a fold change of value assessed in wt mice. To determine the cell numbers, five random regions (0.65 mm^2) were scanned using 20x (NA 0.8) objective and stitched (ZEN black software, Zeiss). The cells were counted manually by a blinded investigator using Cell counter Plugin of Fiji software. Values were expressed as numbers of cells per mm^2.

Translating ribosomal affinity purification (TRAP) and RNA-sequencing. Nine-to-ten week old hemizygous *Snap25l10a* GFP females (B6;FVB-Tg(Snap25-EGFP/Rpl10a)JD362Htz/J; JAX #:030273) were sacrificed with an overdose of isoflurane at 6 dpi. Five cm of distal small intestine was thoroughly flushed with RNAse-free PBS and put on a glass bar. After making a superficial dent with fine forceps, the muscularis externa was isolated. Samples were manually homogenized using a glass dounce tissue grinder (DWK Life Sciences; #8853000007) in homogenization buffer (50 mM Tris, pH 8.0; 100 mM KCl; 12 mM $MgCl_2$; 1 % Igepal; 1 mg/mL sodium heparin; 100 µg/mL cycloheximide; 0.2 U/µL SUPERaseIn RNase Inhibitor, 2 mM DTT). After centrifugation (10,000 x g; 10 min; 4°C) 25 µg of primary antibody against GFP (rabbit; # AB290; Abcam) was added, and samples were rotated for 1 h at 4 °C. Subsequently, 200 µl of Dynabeads™ Protein G (# 10004D; Invitrogen) were added, and samples were incubated rotating overnight at 4 C. The beads were washed four times with high-salt buffer (50 mM Tris, pH 8.0; 300 mM KCl; 12 mM $MgCl_2$; 1 % Igepal; 100 µg/mL cycloheximide). RNA was extracted and purified with Arcturus PicoPure RNA Isolation Kit (ThermoFisher: Kit0204 - Applied biosystems; 12204-01) according to the manufacturer's instructions. To prevent contamination with DNA, RNase-Free DNase Set (Qiagen; # 29254) was used according to the manufacturer's instructions.

RNA integrity was determined using Agilent Bioanalyzer or 4200 Tapestation. Library preparation was performed with 1-50 ng of total RNA. cDNA was prepared using the SeqPlex RNA Amplification Kit (Sigma) per manufacturer's protocol and then fragmented using a Covaris E220 sonicator using peak incident power 18, duty factor 20%, cycles per burst 50 for 120 seconds. cDNA was blunt ended, an A base was added to the 3' ends, and then Illumina sequencing adapters were ligated to the ends. Ligated fragments were then amplified for 12-15 cycles using primers incorporating unique index tags. Fragments were sequenced on an Illumina NovaSeq-6000 using paired end reads extending 150 bases.

Differential gene expression analysis. Sequencing data was demultiplexed using Illumina's DRAGEN and BCLconvert version 4.2.4 software. Reads were then aligned and mapped to the Ensembl release 101 primary assembly with STAR version 2.7.9a (2). A count matrix containing all expression data was then generated from the number of uniquely aligned unambiguous read using Subread:featureCount version 2.0.3 and isoform expression of known Ensembl transcripts quantified with Salmon version 1.5.2 (3, 4). Sequencing performance was assessed for the total number of aligned reads, total number of uniquely aligned reads, and features detected. Genes with low expression across all libraries were then filtered out and expression data was analyzed using DESeq2 with standard parameters, normalization, and the Benjamini-Hochberg adjustment to estimate the false discovery rate (FDR) and correct for multiple testing (5). Differentially expressed genes were ranked by absolute fold-change in gene expression (cutoff log2 fold change > 1) and filtered by significant FDR-corrected p-values (Padj < 0.05). Pathway analyses of differentially expressed genes were performed using the Gene Ontology (GO) databases (6, 7).

Flow cytometry. Blood was collected by submandibular bleeding, transferred to EDTA coated tubes (BD, # 365974), and erythrocytes were lysed by ACK lysis buffer (GIBCO) for 10 to 15 min at RT. After washing with FACS buffer (PBS supplemented with 2% FBS, 2 mM EDTA), Fc-gamma receptors were blocked (anti-CD16/32; Biolegend, # 101301), cells were stained with

viability dye (Zombie NIR; Biolegend, # 423105) and antibodies against CD45 BUV 395 (BD Bioscience, # 564279), CD11b PE (Biolegend, # 101208) , Ly6G PerCP (Biolegend, # 127616) Cy5.5, Ly6C BV421 (Biolegend, # 128031), and then fixed with 2% PFA. Samples were read on BD Fortessa X-20 and analyzed using FlowJo software (FlowJo LLC). For total cell counts, Trucount Absolute counting tubes were used (BD Biosciences, # 340334) according to the manufacturer's instructions.

Organs were harvested from isoflurane-overdosed mice. Single cell suspensions from spleens, Peyer's patches, and mesenteric lymph nodes were obtained by mashing through a cell strainer (70 μm) with syringe plunger, and erythrocytes in spleens were lysed by ACK lysis buffer (GIBCO) for 3 min on ice and then washed with FACS buffer. The small intestine was harvested, cut into 5 cm pieces, flushed with PBS and put on a glass bar. After making a superficial dent with fine forceps, the muscularis externa was isolated by rolling away with cotton Q-tip. The muscularis externa and the remainder of the gut were cut separately into small pieces (0.5 mm) and incubated with digestion buffer (RPMI 1640 Medium, 10% FBS, 1 mM sodium pyruvate, 1 mM HEPES, MEM Nonessential amino acids, β-mercaptoethanol or HBSS with Mg^{2+}/Ca^{2+} (Gibco, # 24020117), 400 U/mL collagenase D, 2.5 U/mL dispase, 25 mM HEPES, 1 mM sodium pyruvate, 50 μg/mL DNase I) at 37°C on shaker (140 rpm) for 40 min. Tissue was homogenized using an 18G needle, washed with HBSS + 5% FBS, and the cell suspension was filtered through 70 um cell strainer. After washing, the samples were resuspended in 40% Percoll (Cytiva, # 17-0891-01), overlaid with 70% Percoll and centrifuged 850 x g for 20 min at 4°C. The interface was collected and washed with FACS buffer. Fc-gamma receptors were blocked (anti-CD16/32; Biolegend # 101302), and cells were stained with viability dye (LIVE/DEAD Fixable Lime viability kit; ThermoFisher Scientific, # L34989) and antibodies against CD45 (Biolegend, # 160306), CD45.1 (Biolegend, # 110743) CD3 (Biolegend, # 100227, 100206), TCRβ (Biolegend, #109243), TCRγδ (BD Bioscience, #744117), CD4 (Biolegend, # 116027, 300554), CD8a (Biolegend #, 100710, 100706,

155013), TCRvβ 8.1/8.2 (Invitrogen, #11-5813-82), TCRvβ 5.1 (Biolegend, # 139505) Foxp3 Transcription Factor Fixation/Permeabilization kit (eBioscience, # 00-5521-00) was used according to manufacturer's instructions for intracellular staining of perforin (Biolegend # 154306). To assess the total cell counts, Precision Count Beads (Biolegend # 424902) were used. Cells were processed on an Aurora Cytek and analyzed by FlowJo v10 software.

Antibody and tamoxifen treatments. For depletion of T cells, mice were injected via intraperitoneal route with 200 μg of anti-CD4 (InVivoMAb, clone GK1.5, BioXCell # BE0003-1) and/or anti-CD8β (InVivoMAb, Lyt 3.2, clone 53-5.8, BioXCell, # BE0223) or rat isotype control mAb (BioXCell InVivoMAb rat IgG1 anti-horseradish peroxidase, # BE0088; rat IgG2b isotype control, LTF-2, # BE0090) as indicated in the schematics of supplemental Figures. To deplete resident macrophages, mice were injected via intraperitoneal route with 50 mg/kg (1.25 mg) of anti-CSF1R mAb (InVivoMAb, AFS98, BioXCell, # BE0213) or isotype control (rat IgG2a, BioXCell, # BE0089) one day prior to WNV infection and 3 dpi. To decrease monocyte infiltration, mice were injected via intraperitoneal route with 50 μg of anti-CCR2 mAb (clone MC21; (8)) or rat IgG2b isotype control (LTF-2, BioXCell, # BE0090). For combined neutrophil and monocyte depletions, animals were given 250 μg of anti-Ly6G/Ly6C mAb (GR-1, clone NIMP-R14, InVivoMAb, BioXCell, # BE0320) or rat IgG2b isotype control mAb (LTF-2, BioXCell, # BE0090) one day prior to WNV infection and then 2, 4, and 6 dpi. To block IFNγ and TNF, mice were injected via intraperitoneal route with 200 μg of anti-TNF (MP6-XT22, GoInVivo, BioLegend, # 506352) or rat IgG2b isotype control mAb (LTF-2, BioXCell, # BE0090) or anti-IFNγ (Leinco Technologies, # I-438) or Armenian hamster IgG isotype control (Leinco Technologies, # 1196) one day prior to infection and at 2 and 5 dpi.

Tamoxifen (Sigma, # T5648) was resuspended in corn oil and 250 μl of a 20 mg/mL solution was administered via oral gavage to *Ccr2* CreER YFP mice 1 day prior to WNV infection and then at 3, 7 and 11 dpi.

Supplemental Figures

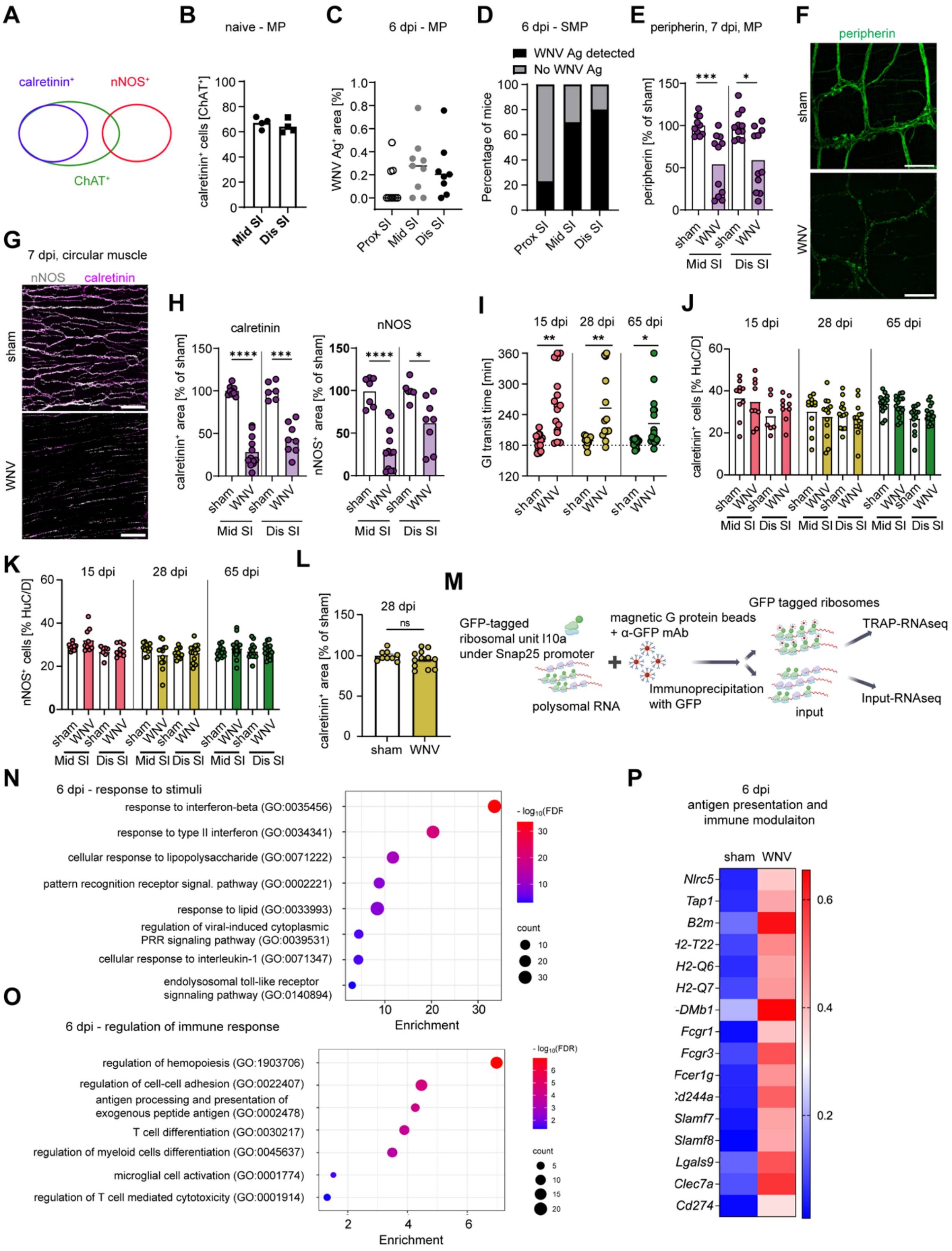

Supplemental Figure S1. WNV tropism and effects on neurons. Related to Figure 1. (**A**) Co-expression of neuronal markers in the myenteric plexus. The diagram shows that most $ChAT^+$ cells also express calretinin and some nNOS. (**B**) Whole mounts of middle and distal SI from sham-infected *ChAT*$^+$ GFP mice after staining for calretinin. Most neurons (70%) co-express both calretinin and ChAT, the residual 30% of neurons express only ChAT; these cells account for the higher percentage of WNV infected cells shown in Figure 1C. (**C, D**) Whole mount preparations of muscularis externa from proximal, mid, and distal SI were isolated at 6 dpi and stained for WNV antigen. (**C**) Data are presented as percentage of WNV antigen positive area in the MP at 6 dpi. (**D**) Muscularis externa was isolated with layer containing submucosal plexus (SMP) at 6 dpi. Data are presented as the percentage of mice with WNV antigen in the SMP. (**E-H, J-L**) Whole mount preparations of muscularis externa from (**E-H**) middle and distal SI or (**L**) proximal SI were isolated at (**E-H**) 7 dpi or (**L**) 28 dpi and stained for (**E, F**) peripherin, (**G, H**) calretinin and nNOS, or (**L**) calretinin. The fraction of area staining positive for neuronal markers in the (**E**) MP or (**H**) circular muscle was determined, and the values were normalized to sham-infected mice. Representative images show staining in mid SI in sham and WNV-infected mice at 7 dpi. Scale bar; 100 μm. (**I**) GI transit time in sham or WNV-infected mice at indicated time points. (**J-K**) Muscularis externa was isolated from the mid and distal regions of SI of sham or WNV-infected mice at 15, 28 or 65 dpi and stained for neuronal markers HuC/D, nNOS, and calretinin. Data are shown as percentage of HuC/D^+ neurons. (**M**) Scheme of Translating Ribosome Affinity Purification (TRAP) used for enrichment of RNA from enteric neurons in muscularis externa. (**N-P**) RNAseq analysis of neuron-enriched samples from sham or WNV-infected *Snap25l10a* GFP mice 6 dpi. (**N, O**) Bubble plots of GO pathways represented by differentially expressed genes in sham and WNV-infected mice involved in (**N**) response to stimuli or (**O**) regulation of immune response. (**P**) Heatmap of differentially expressed genes involved in antigen presentation and immune modulation. Gene expression is normalized across each gene and represent the average of 4 samples per condition. Data are pooled from the following number of experiments (left to right): (**B**) 2; (**C**) 2; (**D**) 3; (**E**) 3; (**H**) 3; (**I**) 3, 2, 3; (**J, K**) 2, 3, 4; (**L**) 2; (**N-P**) 1. The indicated numbers of mice per group are shown from left to right: (**B**) 4, 4; (**C**) 9, 9, 9; (**D**) 10, 11, 11, 11; (**E**) 9, 12, 6, 8, 7, 12, 6, 8; (**I**) 17, 12, 13, 13, 1—7; (**J, K**) 10, 9, 7, 9, 13, 13, 13, 13, 14, 16, 14, 16; (**L**) 9, 12; (**N, P**) 4. Lines and column heights indicate mean values. Statistical analysis: (**E, H, I, J, K**) two-tailed Mann-Whitney test: not significant, ns, *$p < 0.05$, **$p < 0.01$.

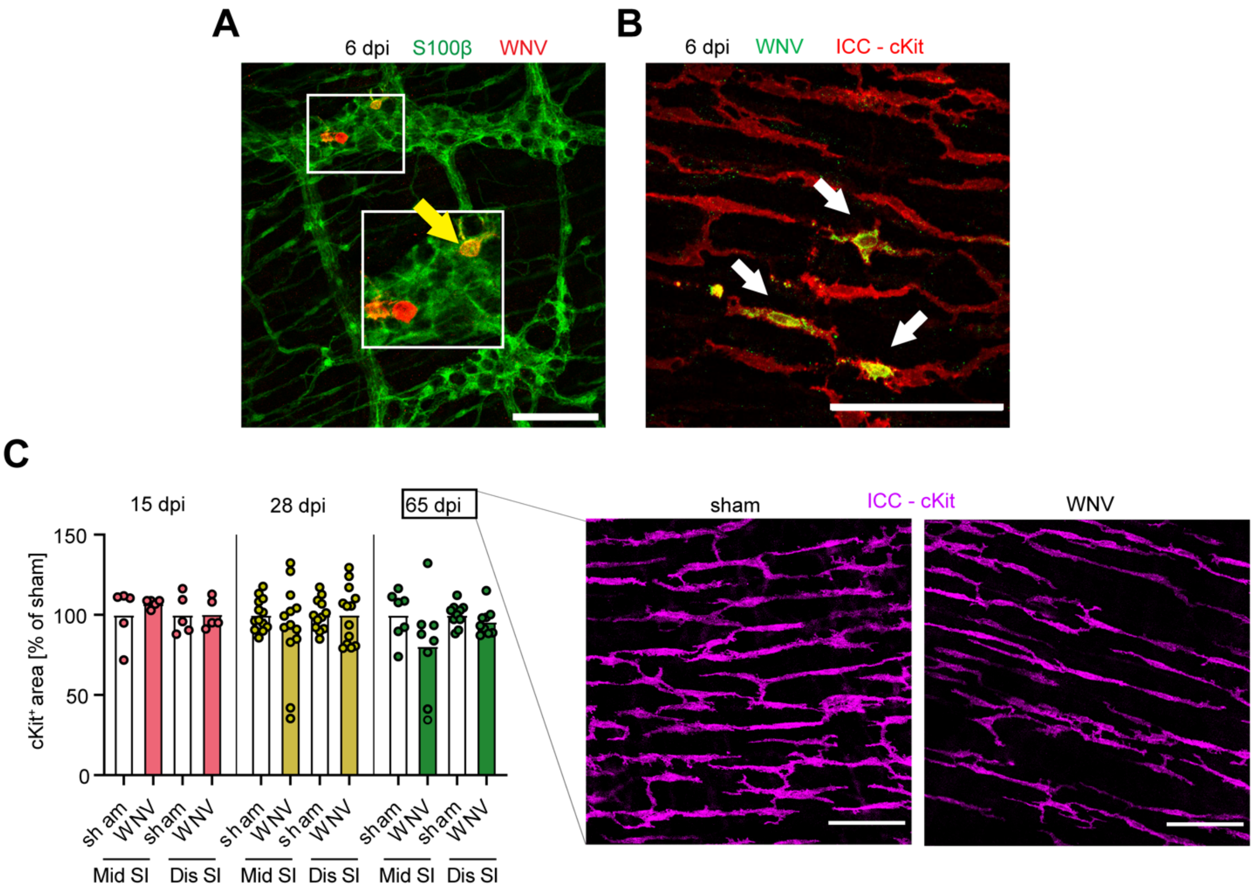

Supplemental Figure S2. Effect of WNV infection on ICCs, Related to Figure 2. (**A-C**) Muscularis externa was isolated from the middle or distal regions of small intestine of sham or WNV-infected wt mice at (**A, B**) 6 dpi or (**C**) 15, 28, or 65 dpi and stained for (**A**) glial marker S100β and WNV antigen or (**B**) ICC marker cKit and WNV antigen. Scale bar, 100 μm. (**C**) The fraction of area staining positive for cKit in the circular muscle layer was determined, and the values were normalized to sham-infected mice. Representative images are from at least 2 experiments. (**B**) White arrows indicate WNV-infected ICCs. Column heights indicate mean values. Data are from (**D**) from left to right: 1, 3, 3 experiments, n = 5, 5, 5, 5, 13, 13, 13, 12, 13, 7, 8, 10, 9 mice.

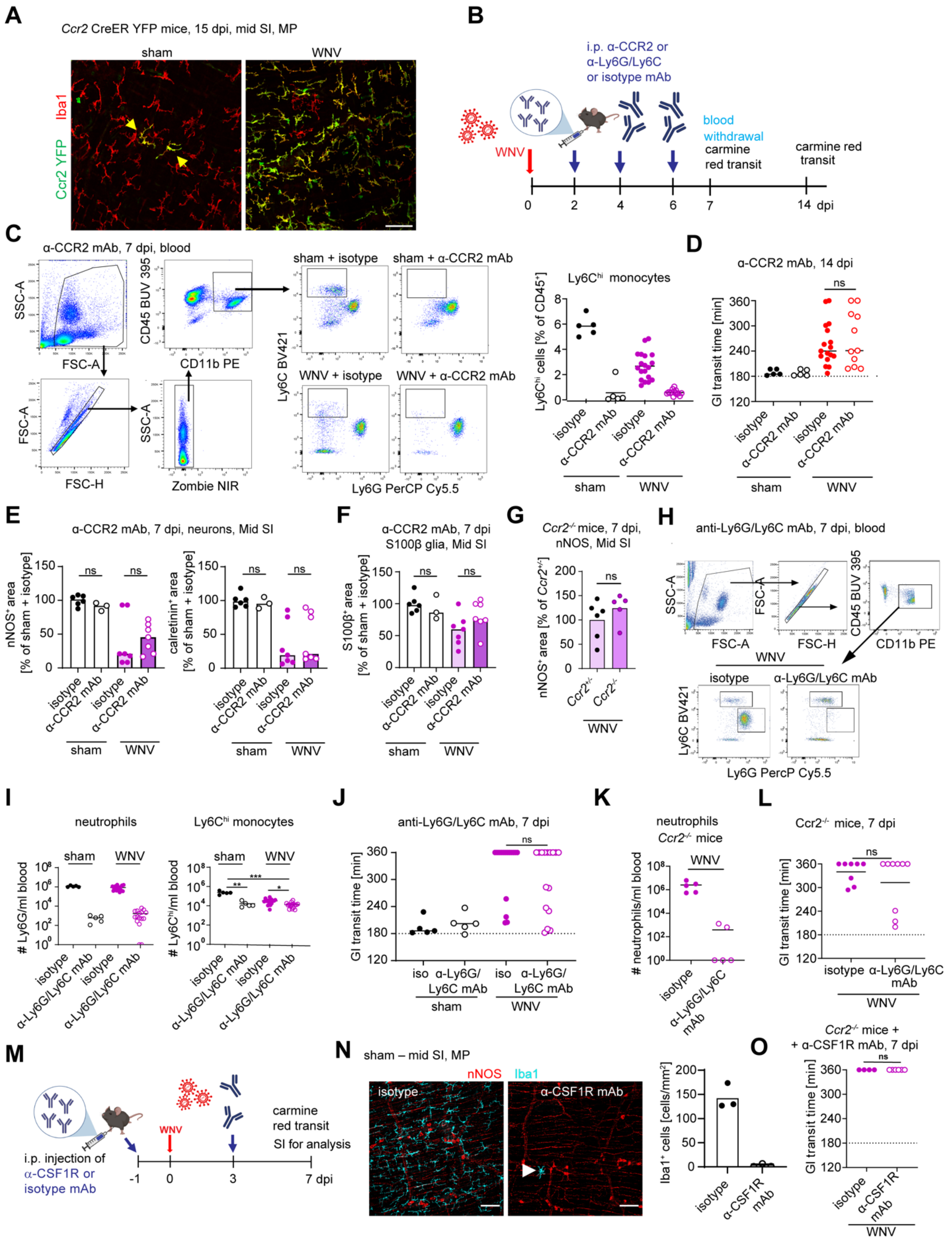
A
Ccr2 CreER YFP mice, 15 dpi, mid SI, MP
sham
WNV
Iba1
Ccr2 YFP
B
i.p. α-CCR2 or α-Ly6G/Ly6C or isotype mAb
WNV
blood withdrawal
carmine red transit
carmine red transit
0 2 4 6 7 14 dpi
C
α-CCR2 mAb, 7 dpi, blood
SSC-A
FSC-A
FSC-A
FSC-H
CD45 BUV 395
CD11b PE
SSC-A
Zombie NIR
sham + isotype
sham + α-CCR2 mAb
WNV + isotype
WNV + α-CCR2 mAb
Ly6C BV421
Ly6G PercP Cy5.5
Ly6Chi monocytes
Ly6Chi cells [% of CD45+]
isotype
α-CCR2 mAb
sham
WNV
D
α-CCR2 mAb, 14 dpi
GI transit time [min]
ns
E
α-CCR2 mAb, 7 dpi, neurons, Mid SI
nNOS+ area [% of sham + isotype]
calretinin+ area [% of sham + isotype]
F
α-CCR2 mAb, 7 dpi S100β glia, Mid SI
S100β+ area [% of sham + isotype]
G
Ccr2-/- mice, 7 dpi, nNOS, Mid SI
nNOS+ area [% of Ccr2+/-]
Ccr2+/-
Ccr2-/-
H
anti-Ly6G/Ly6C mAb, 7 dpi, blood
WNV
isotype
α-Ly6G/Ly6C mAb
I
neutrophils
Ly6Chi monocytes
Ly6G/ml blood
Ly6Chi/ml blood
α-Ly6G/Ly6C mAb
J
anti-Ly6G/Ly6C mAb, 7 dpi
iso
α-Ly6G/Ly6C mAb
K
neutrophils Ccr2-/- mice
neutrophils/ml blood
L
Ccr2-/- mice, 7 dpi
M
i.p. injection of α-CSF1R or isotype mAb
carmine red transit
SI for analysis
-1 0 3 7 dpi
N
sham – mid SI, MP
nNOS Iba1
isotype
α-CSF1R mAb
Iba1+ cells [cells/mm2]
O
Ccr2-/- mice + α-CSF1R mAb, 7 dpi

Supplemental Figure S3. Infiltrating monocytes and macrophages do not contribute to WNV-induced damage to neurons and glia and ensuing GI dysmotility, Related to Figure 3. (**A, E, F, G, N**) Muscularis externa was isolated from the (**A, G, N**) middle or (**E, F**) middle and distal region of small intestine (SI) of (**A**) sham or WNV-infected *Ccr2* CreER YFP mice at 15 dpi, (**E, F**) sham or WNV-infected wt mice treated with anti-CCR2 Ab at 7 dpi, (**G**) WNV-infected Ccr2$^{-/+}$ and Ccr2$^{-/-}$ mice at 7 dpi, or (**N**) sham mice treated with anti-CSF1R mAb and stained for (**A**) macrophage marker Iba1, (**E, F**) calretinin and nNOS, (**G**) nNOS, or (**N**) Iba1 and nNOS. (**A**) Representative images are from the middle region of SI of 3 mice per group. Scale bar, 100 μm. (**E, F, G**) The fraction of positive area for (**E**) calretinin and nNOS, (**F**) S100β, (**G**) nNOS in the MP was determined, and values were normalized to (**E, F**) sham-infected wt mice treated with isotype mAb or (**G**) to WNV-infected *Ccr2*$^{+/-}$ mice. (**N**) Iba1^{+} cells were counted in the MP, and cell counts are expressed as the number of Iba1^{+} cells per mm^{2}. (**B**) Experimental design of monocyte/neutrophil depletion after WNV infection using anti-CCR2 or Ly6G/Ly6C mAb treatments. GI transit was assessed at 7 dpi (anti-CCR2 and anti-Ly6G/Ly6C and treated groups) or 14 dpi (only CCR2 mAb-treated group). Blood was obtained at 7 dpi. (**C, H, I, K**) Leukocytes from sham or WNV-infected (**C, H, I**) wt or (**K**) *Ccr2*$^{-/-}$ mice treated with (**C**) anti-CCR2 mAb or (**H, I, K**) anti-Ly6G/Ly6C mAb were stained with antibodies to CD45, CD11b, Ly6G, and Ly6C. (**C**) Percentages and (**I, K**) numbers (**C**) of monocytes (CD45^{+} CD11b^{+} Ly6G^{-} Ly6C^{hi}) and/or (**I, K**) neutrophils (CD45^{+} CD11b^{+} Ly6G^{+} Ly6C^{+}). (**C, H**). Gating strategy of (**C**) monocyte and (**H**) monocyte/neutrophil populations by flow cytometry. (**D, J, L, O**) GI transit was measured in (**D**) in sham or WNV-infected mice after treatment with anti-CCR2 or isotype mAbs at 14 dpi, (**J**) in sham or WNV-infected mice after treatment with anti-Ly6G/Ly6C, or isotype control mAb at 7 dpi, (**L**) WNV-infected *Ccr2*$^{-/-}$ mice treated with anti-Ly6G/Ly6C or isotype control mAb at 7 dpi or (**O**) WNV infected *Ccr2*$^{-/-}$ mice treated with anti-CSF1R or isotype mAb at 7 dpi. Data are pooled from the following number of experiments: (**C, D, H, G**) 3; (**E, F, L, N, O**) 2; and (**I**) 1. The indicated numbers of mice per group are shown from left to right: (**C**) 5, 5, 20,15; (**D**) 5, 5, 17, 11; (**E, F**) 6, 3, 7, 7; (**G**) 6, 5; (**I, J**) 5, 5, 20, 15; (**K**) 5, 5; (**L**) 8, 9; (**N**) 3, 3 and (**O**) 4, 6. Lines and column heights indicate mean values. Statistical analysis: (**D, E, F, G, K, L, O**) two-tailed Mann-Whitney test; (**I**) Kruskal-Wallis ANOVA with Dunn's post-test. Not significant, ns, *p < 0.05, **p < 0.01, ***p < 0.001.

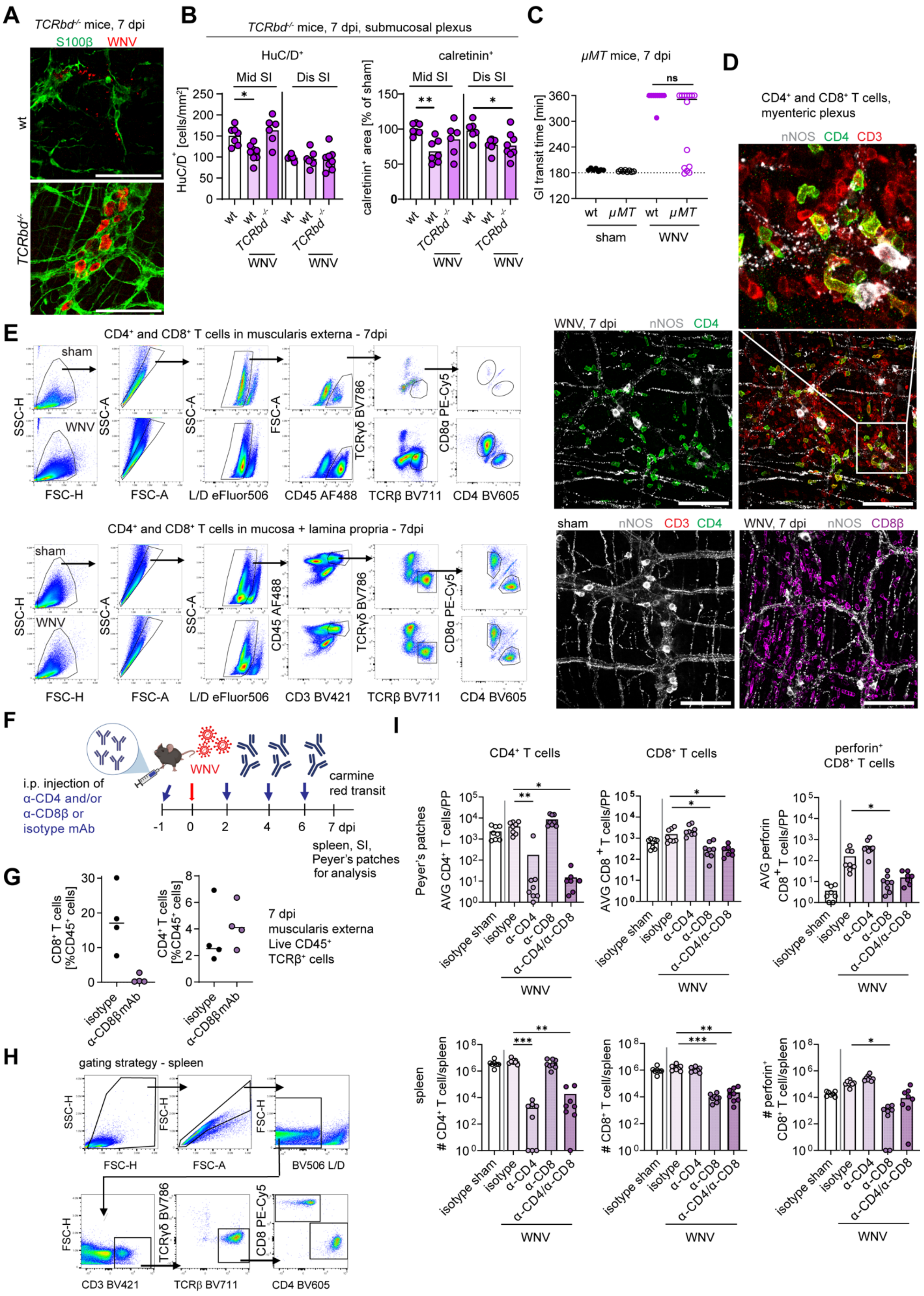
A
TCRbd-/- mice, 7 dpi
S100β WNV
wt
TCRbd-/-
B
TCRbd-/- mice, 7 dpi, submucosal plexus
HuC/D+
Mid SI
Dis SI
calretinin+
HuC/D+ [cells/mm2]
calretinin+ area [% of sham]
wt
TCRbd-/-
WNV
C
μMT mice, 7 dpi
GI transit time [min]
ns
sham
WNV
D
CD4+ and CD8+ T cells, myenteric plexus
nNOS CD4 CD3
WNV, 7 dpi nNOS CD4
sham nNOS CD3 CD4
WNV, 7 dpi nNOS CD8β
E
CD4+ and CD8+ T cells in muscularis externa - 7dpi
sham
WNV
SSC-H
SSC-A
FSC-A
TCRγδ BV786
CD8α PE-Cy5
FSC-H
L/D eFluor506
CD45 AF488
TCRβ BV711
CD4 BV605
CD4+ and CD8+ T cells in mucosa + lamina propria - 7dpi
CD45 AF488
CD3 BV421
F
i.p. injection of α-CD4 and/or α-CD8β or isotype mAb
WNV
carmine red transit
-1 0 2 4 6 7 dpi
spleen, SI, Peyer's patches for analysis
G
CD8+ T cells [%CD45+ cells]
CD4+ T cells [%CD45+ cells]
isotype
α-CD8βmAb
7 dpi
muscularis externa
Live CD45+
TCRβ+ cells
H
gating strategy - spleen
SSC-H
FSC-H
FSC-A
BV506 L/D
CD3 BV421
TCRγδ BV786
TCRβ BV711
CD8 PE-Cy5
CD4 BV605
I
CD4+ T cells
CD8+ T cells
perforin+ CD8+ T cells
Peyer's patches
AVG CD4+ T cells/PP
AVG CD8+ T cells/PP
AVG perforin CD8+ T cells/PP
spleen
CD4+ T cell/spleen
CD8+ T cell/spleen
perforin+ CD8+ T cell/spleen
isotype sham
isotype
α-CD4
α-CD8
α-CD4/α-CD8
WNV

Supplemental Figure S4. CD4$^+$ and CD8$^+$ T cells contribute to WNV-triggered ENS injury and GI dysmotility, Related to Figure 4. (**A, B, D**) Whole mount preparations of muscularis externa were isolated from WNV-infected wt and *TCRbd*$^{-/-}$ mice at 7 dpi and co-stained for (**A**) WNV antigen and glia (S100β), (**B**) HuC/D and calretinin or (**D**) T cell markers CD3, CD4 or CD8, and nNOS. Representative images from (**A**) 2 experiments or (**D**) of at least 3 mice, scale bar, 100 μm. (**B**) The fraction of positive area for calretinin in the SMP was determined, and values were normalized to sham-infected wt mice. HuC/D$^+$ cells were counted in the SMP, and cell counts are expressed as the number of HuC/D$^+$ cells per mm^2. Values for wt mice are shown for comparison and are identical to those shown in Figure 1E. (**C**) GI transit time of sham or WNV-infected wt or *μMT* mice at 7 dpi. (**E, G**) Flow cytometric analysis of small intestine at 7 dpi. (**E**) Gating strategy and (**G**) proportions of CD4$^+$ and CD8$^+$ T cell populations in (**E**) muscularis externa or mucosa and lamina propria (residual) or (**G**) muscularis externa of wt mice treated with anti-CD8β mAb. (**F**) Experimental design of T cell depletions. Mice were injected with anti-CD4, anti-CD8β, or both anti-CD4 and CD8 mAbs. (**H, I**) CD4$^+$ and CD8$^+$ T cells (**G**) in muscularis externa or (**I**) in spleen and Peyer's patches harvested from sham or WNV-infected mice treated with anti-CD4/CD8 or isotype mAb and stained for CD45, CD3, TCRβ, TCRγδ, CD4, CD8α, and perforin and gated on (**G**) live CD45$^+$ TCRβ$^+$ TCRγδ$^-$ or (**I**) live CD3$^+$ TCRβ$^+$ TCRγδ$^-$ CD4$^+$ or CD8$^+$ T cells (shown in **H**). Data are shown as (**G**) percentage of CD45$^+$ cells in SI and (**I**) total cell numbers per spleen and average cell numbers per Peyer's patch. Data are pooled from the following number of experiments: (**B**) 3, (**C**) 3, (**G**) 1, (**I**) 2. The indicated numbers of mice are shown per group (left to right): (**B**) 7, 8, 6, 6, 7, 8, 7, 7, 6, 6, 7, 8; (**C**) 7, 8, 9, 14; (**G**) 4, 4, 4, 4; (**I**); 8, 8, 8, 8, 7 (Peyer's patches), 7, 8, 8, 8, 8 (spleen). Lines indicate median values. Column heights indicate mean values. Statistical analysis: (**C**) two-tailed Mann-Whitney test; (**B, H**) Kruskal-Wallis ANOVA with Dunn's post-test comparisons were to "wt sham" (**B**) or "isotype WNV control" (**H**) groups: not significant, ns, *p < 0.05, **p < 0.01, ***p < 0.001.

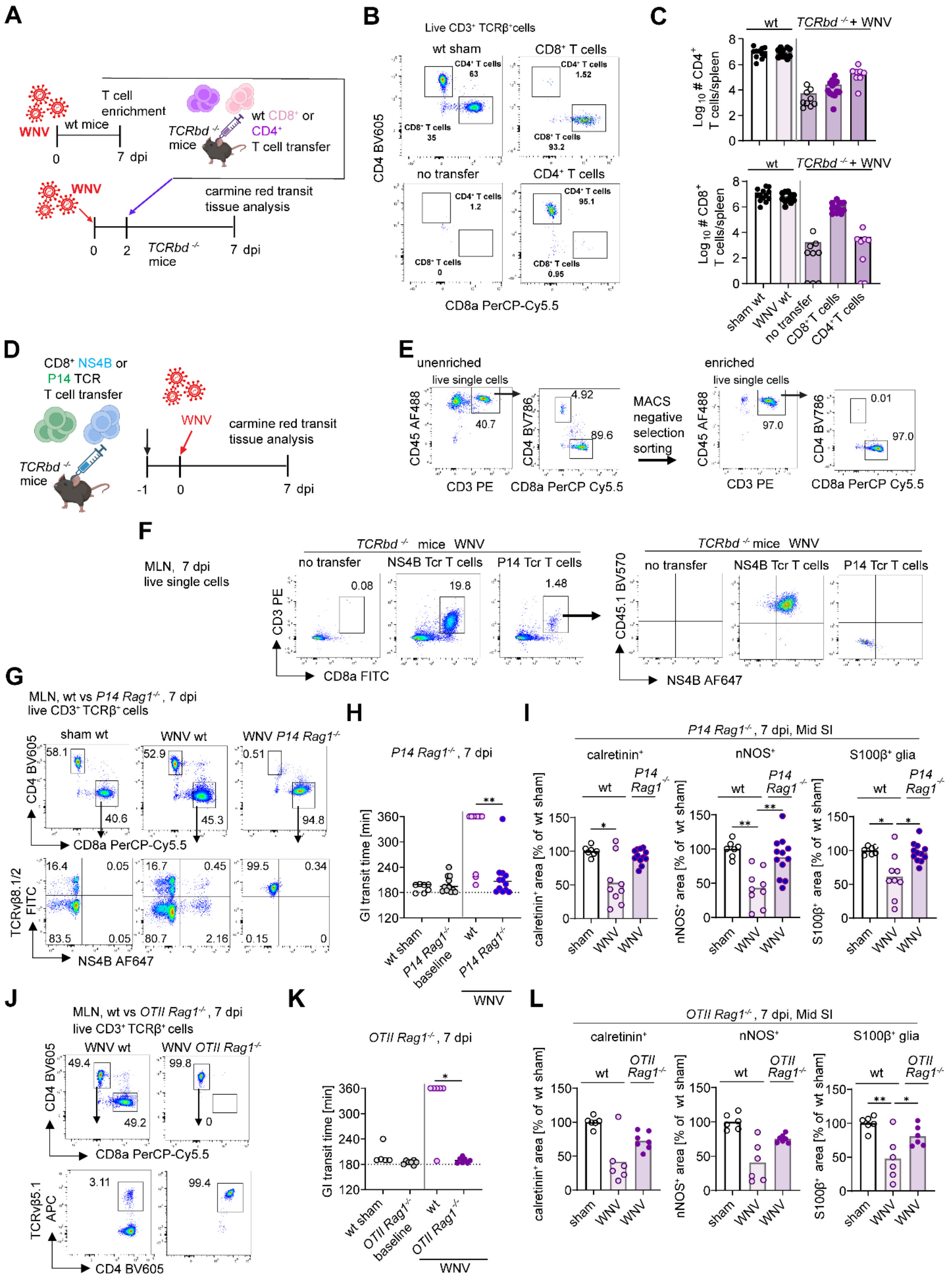
A
T cell enrichment
WNV
wt mice
0
7 dpi
TCRbd -/- mice
wt CD8+ or CD4+ T cell transfer
carmine red transit tissue analysis
0
2
7 dpi
B
Live CD3+ TCRβ+cells
wt sham
CD8+ T cells
no transfer
CD4+ T cells
CD4 BV605
CD8a PerCP-Cy5.5
C
wt
TCRbd -/- + WNV
Log 10 # CD4+ T cells/spleen
Log 10 # CD8+ T cells/spleen
sham wt
WNV wt
no transfer
CD8+ T cells
CD4+ T cells
D
CD8+ NS4B or P14 TCR T cell transfer
TCRbd -/- mice
WNV
carmine red transit tissue analysis
-1
0
7 dpi
E
unenriched
enriched
live single cells
CD45 AF488
CD3 PE
CD4 BV786
CD8a PerCP Cy5.5
MACS negative selection sorting
F
MLN, 7 dpi live single cells
TCRbd -/- mice WNV
no transfer
NS4B Tcr T cells
P14 Tcr T cells
CD3 PE
CD8a FITC
CD45.1 BV570
NS4B AF647
G
MLN, wt vs P14 Rag1-/-, 7 dpi live CD3+ TCRβ+ cells
sham wt
WNV wt
WNV P14 Rag1-/-
CD4 BV605
CD8a PerCP-Cy5.5
TCRvβ8.1/2 FITC
NS4B AF647
H
P14 Rag1-/-, 7 dpi
GI transit time [min]
wt sham
P14 Rag1-/- baseline
wt
P14 Rag1-/-
WNV
I
P14 Rag1-/-, 7 dpi, Mid SI
calretinin+
nNOS+
S100β+ glia
calretinin+ area [% of wt sham]
nNOS+ area [% of wt sham]
S100β+ area [% of wt sham]
sham
WNV
J
MLN, wt vs OTII Rag1-/-, 7 dpi live CD3+ TCRβ+ cells
WNV wt
WNV OTII Rag1-/-
CD4 BV605
CD8a PerCP-Cy5.5
TCRvβ5.1 APC
CD4 BV605
K
OTII Rag1-/-, 7 dpi
GI transit time [min]
wt sham
OTII Rag1-/- baseline
wt
OTII Rag1-/-
WNV
L
OTII Rag1-/-, 7 dpi, Mid SI
calretinin+
nNOS+
S100β+ glia
sham
WNV

Supplemental Figure S5. Damage to neuronal and glial network is caused by WNV-specific $CD4^+$ and $CD8^+$ T cells, Related to Figure 5. (**A**) Experimental design of adoptive transfer of wt $CD8^+$ or $CD4^+$ T cells to *TCRbd*$^{-/-}$ mice. $CD8^+$ (5×10^6) or $CD4^+$ (10^7) T cells from wt spleens and MLNs at 7 dpi were enriched and injected intravenously into WNV-infected *TCRbd*$^{-/-}$ mice at 2 dpi. (**B, C**) Flow cytometric analysis of splenocytes from *TCRbd*$^{-/-}$ mice after transfer of primed wt T cells at 7 dpi. Spleens were harvested and stained for CD3, TCRβ, TCRγδ, CD4 and CD8α and gated on live $CD3^+$ $TCR\beta^+$ $TCR\gamma\delta^-$ $CD4^+$ or $CD8^+$ T cells. (**B**) Representative flow cytometry dot plots and (**C**) total cell numbers per spleen. (**D**) *TCRbd*$^{-/-}$ mice were adoptively transferred by intravenous injection with 10^6 WNV NS4B or LCMV P14 gp33 transgenic $CD8^+$ T cells and then inoculated subcutaneously with WNV one day later. (**E, F**) Representative flow cytometry dot plots of (**E**) splenocytes before and after enrichment for NS4B and LCMV P14 gp33 transgenic $CD8^+$ T cells and (**F**) of T cells from mesenteric lymph nodes of *TCRbd*$^{-/-}$ mice injected with NS4B or P14 transgenic T cells at 7 dpi. Cells were stained with mAbs against (**E**) CD45, CD3, CD4 and CD8α and gated on live $CD45^+$ $CD3^+$ $CD4^+$ or $CD8^+$ T cells or (**F**) CD45.1 (to identify NS4B transgenic $CD8^+$ T cells), CD3, CD8a, and WNV NS4B D^b-restricted tetramers and gated on live cells. (**G, J**) Flow cytometric analysis of cells in MLNs from WNV-infected wt or (**G**) wt sham or WNV-infected *P14 Rag1*$^{-/-}$ mice or (**J**) WNV-infected *OT-II Rag1*$^{-/-}$ mice at 7 dpi. MLNs were harvested at 7 dpi and stained for CD3, TCRβ, TCRγδ, CD4, CD8α and (**G**) TCRvβ 8.1/8.2 specific for LCMV and WNV NS4B D^b-restricted tetramers or (**J**) TCR vβ 5.1 specific for OVA and gated on live $CD3^+$ $TCR\beta^+$ $TCR\gamma\delta^-$ $CD4^+$ or $CD8^+$ T cells. (**H, K**) GI transit was measured at 7 dpi. Transit time of (**H**) sham-infected or WNV-infected wt or *P14 Rag1*$^{-/-}$ mice or (**K**) WNV-infected *OT-II Rag1*$^{-/-}$ mice. (**I, L**) Muscularis externa was isolated from the mid regions of small intestine (SI) of (**I**) sham-infected or WNV-infected wt or *P14 Rag1*$^{-/-}$ mice or (**L**) WNV-infected *OT-II Rag1*$^{-/-}$ mice at 7 dpi and stained. The fraction of area positive for calretinin, nNOS or S100β was determined, and values were normalized to wt sham mice. Data are pooled from (**C, G, H, I**) 3 and (**J, K, L**) 2 experiments with indicated numbers of mice per group (left to right): (**C**) 4, 5, 4, 4, 4; (**H**) 7, 10, 12, 12; (**I**) 7, 9, 12, 7, 9, 12, 7, 9, 12; (**K**) 5, 6, 9, 9; (**L**) 6, 6, 7, 7. Representative flow plots are from (**E, F, G**) 3 or (**J**) 2 experiments. Lines and column heights indicate the mean values. Statistical analysis: (**H, K**) two-tailed Mann-Whitney test; (**I, L**) Kruskal-Wallis one-way ANOVA with Dunn's post-test: not significant, ns, *$p < 0.05$, **$p < 0.01$, ***$p < 0.001$.

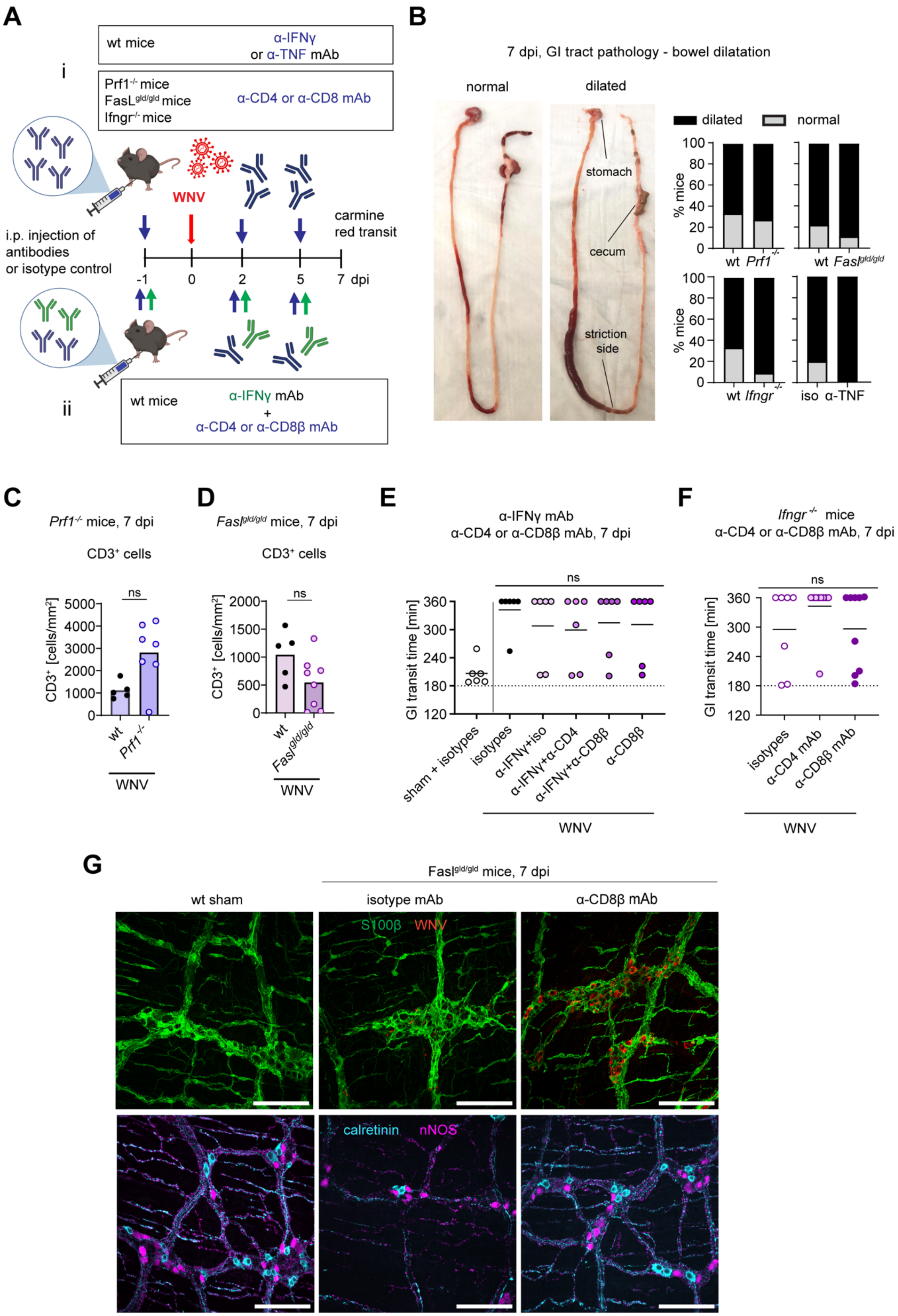
A
i
wt mice
α-IFNγ or α-TNF mAb
Prf1-/- mice
FasLgld/gld mice
Ifngr-/- mice
α-CD4 or α-CD8 mAb
WNV
i.p. injection of antibodies or isotype control
carmine red transit
-1 0 2 5 7 dpi
ii
wt mice
α-IFNγ mAb + α-CD4 or α-CD8β mAb
B
7 dpi, GI tract pathology - bowel dilatation
normal
dilated
stomach
cecum
striction side
dilated
normal
% mice
wt Prf1-/-
wt Faslgld/gld
wt Ifngr-/-
iso α-TNF
C
Prf1-/- mice, 7 dpi
CD3+ cells
CD3+ [cells/mm2]
ns
wt
Prf1-/-
WNV
D
Faslgld/gld mice, 7 dpi
CD3+ cells
CD3+ [cells/mm2]
ns
wt
Faslgld/gld
WNV
E
α-IFNγ mAb
α-CD4 or α-CD8β mAb, 7 dpi
GI transit time [min]
ns
sham + isotypes
isotypes
α-IFNγ+iso
α-IFNγ+α-CD4
α-IFNγ+α-CD8β
α-CD8β
WNV
F
Ifngr-/- mice
α-CD4 or α-CD8β mAb, 7 dpi
GI transit time [min]
ns
isotypes
α-CD4 mAb
α-CD8β mAb
WNV
G
Faslgld/gld mice, 7 dpi
wt sham
isotype mAb
α-CD8β mAb
S100β WNV
calretinin nNOS

Supplemental Figure S6. Damage to neuronal and glial network is caused by multiple effector functions of T cells, Related to Figures 6 and 7. (**A**) Experimental design of (**i**) T cell depletion in *Prf1$^{-/-}$, Fasl$^{gld/gld}$, Ifngr$^{-/-}$* mice or IFNγ or TNF blockade in wt mice and (**ii**) combined T cell depletion and IFNγ blockade in wt mice after WNV infection using anti-CD4, anti-CD8β, and anti-IFNγ mAb treatments as indicated. GI transit was assessed at 7 dpi. (**B**) Proportions of wt or *Prf1$^{-/-}$, Fasl$^{gld/gld}$, Ifngr$^{-/-}$* or wt mice treated with anti-TNF blocking mAb or isotype control mAb with abnormally dilated bowel at 7 dpi. (**C, D**) Whole mount preparations of muscularis externa were isolated from WNV-infected wt and (**C**) *Prf1$^{-/-}$* or (**D**) *Fasl$^{gld/gld}$* mice at 7 dpi and stained for T cell marker CD3. Numbers of CD3^{+} cells in the myenteric plexus were calculated by dividing the area positive for CD3^{+} staining with average size of CD3^{+} cell. Cell counts are expressed as numbers of CD3^{+} cells per mm^{2}. (**E, F**) GI tract transit was measured at 7 dpi in wt mice treated with (**E**) anti-IFNγ in combination with anti-CD4 or anti-CD8β mAb or isotype control mAb or (**F**) in *Ifngr$^{-/-}$* mice treated with anti-CD4 or anti-CD8β mAb or isotype control mAb. (**G**) Representative staining images of the myenteric plexus are from middle region of small intestine (SI) in wt sham, WNV-infected *Fasl$^{gld/gld}$* mice treated with anti-CD8β or isotype mAb at 7 dpi. Scale bar, 100 µm. Data are pooled from (**B**) 2 , (**C, D**) 2,(**E**) 2, (**F**) 3 experiments with indicated numbers of mice per group (left to right): (**B**) 5, 11, 9, 9, 10, 10; (**C**) 5, 7; (**D**) 5, 8 ;(**E**) 6, 6, 6, 6, 6, 6 (**F**) 7, 9, 9. (**G**) Representative images of 3 experiments. . Lines and column heights indicate the mean values. Statistical analysis: (C, D) two-tailed Mann-Whitney test; (**E, F**) ANOVA with Dunnett's post-test (comparison to "WNV isotype control" group): not significant, ns.

Supplemental References

1. Diamond MS, Shrestha B, Marri A, Mahan D, and Engle M. B cells and antibody play critical roles in the immediate defense of disseminated infection by West Nile encephalitis virus. *J Virol.* 2003;77(4):2578-86.
2. Dobin A, Davis CA, Schlesinger F, Drenkow J, Zaleski C, Jha S, et al. STAR: ultrafast universal RNA-seq aligner. *Bioinformatics.* 2013;29(1):15-21.
3. Liao Y, Smyth GK, and Shi W. featureCounts: an efficient general purpose program for assigning sequence reads to genomic features. *Bioinformatics.* 2014;30(7):923-30.
4. Patro R, Duggal G, Love MI, Irizarry RA, and Kingsford C. Salmon provides fast and bias-aware quantification of transcript expression. *Nat Methods.* 2017;14(4):417-9.
5. Love MI, Huber W, and Anders S. Moderated estimation of fold change and dispersion for RNA-seq data with DESeq2. *Genome Biol.* 2014;15(12):550.
6. Ashburner M, Ball CA, Blake JA, Botstein D, Butler H, Cherry JM, et al. Gene ontology: tool for the unification of biology. The Gene Ontology Consortium. *Nat Genet.* 2000;25(1):25-9.
7. Aleksander SA, Balhoff J, Carbon S, Cherry JM, Drabkin HJ, Ebert D, et al. The Gene Ontology knowledgebase in 2023. *Genetics.* 2023;224(1).
8. Mack M, Cihak J, Simonis C, Luckow B, Proudfoot AE, Plachý J, et al. Expression and characterization of the chemokine receptors CCR2 and CCR5 in mice. *J Immunol.* 2001;166(7):4697-704.
